# Plant growth and fertility requires functional interactions between specific PABP and eIF4G gene family members

**DOI:** 10.1371/journal.pone.0191474

**Published:** 2018-01-30

**Authors:** Daniel R. Gallie

**Affiliations:** Department of Biochemistry, University of California, Riverside, CA, United States of America; John Curtin School of Medical Research, AUSTRALIA

## Abstract

The initiation of protein synthesis requires the involvement of the eukaryotic translation initiation factor (eIF) 4G to promote assembly of the factors needed to recruit a 40S ribosomal subunit to an mRNA. Although many eukaryotes express two eIF4G isoforms that are highly similar, those in plants, referred to as eIF4G and eIFiso4G, are highly divergent in size, sequence, and domain organization. Species of the Brassicaceae and the Cleomaceae also express a divergent eIFiso4G isoform, referred to as eIFiso4G2, not found elsewhere in the plant kingdom. Despite their divergence, eIF4G and eIFiso4G interact with eIF4A, eIF4B, and eIF4E isoforms needed for binding an mRNA. eIF4G and eIFiso4G also interact with the poly(A)-binding protein (PABP) which promotes ribosome recruitment to an mRNA. Increasing the complexity of such an interaction, however, *Arabidopsis* also expresses three PABP isoforms (PAB2, PAB4, and PAB8) in vegetative and reproductive tissues. In this study, the functional interactions among the eIF4G and the widely-expressed PABP isoforms were examined. Loss of PAB2 or PAB8 in combination with loss of eIF4G or eIFiso4G had little to no effect on growth or fertility whereas *pab2 pab8 eif4g* or *pab2 pab8 eifiso4g1/2* mutants exhibited smaller stature and reduced fertility. Although the *pab4 eifiso4g1* mutant grows normally and is fertile, *pab4 eif4g* or *pab4 eifiso4g2* mutants could not be isolated. Even *pab4/PAB4 eif4g/eIF4G* heterozygous plants exhibited growth defects and low fertility. Mutant co-inheritance analysis in reciprocal crosses with wild-type plants revealed that most ovaries and pollen from *pab4/PAB4 eif4g/eIF4G* plants were *PAB4 eif4g*. Similarly, co-inheritance studies with *pab4/PAB4 eifiso4g2/eIFiso4G2* plants suggested most ovaries were *PAB4 eifiso4g2*. These results suggest that a functional interaction between PAB4 and eIF4G and between PAB4 and eIFiso4G2 is required for growth and normal fertility.

## Introduction

Protein synthesis in eukaryotes requires multiple translation initiation factors that promote 40 S ribosomal subunit recruitment to an mRNA, initiation codon recognition, and 80 S ribosome assembly [1, 2, 3]. One such eukaryotic initiation factor (eIF) is eIF4F composed of eIF4E, which binds the 5′-cap structure; eIF4A which is an RNA helicase involved in unwinding secondary structure in a 5′-leader that might impede 40 S subunit scanning; and eIF4G, which interacts with eIF4E, eIF4A, eIF4B (which stimulates the RNA helicase activity of eIF4A), eIF3 (required for 40 S binding to an mRNA), and the poly(A)-binding protein (PABP) [2, 4, 5, 6]. The eIF4E-eIF4G interaction and the eIF4G-PABP interaction bridge the termini of an mRNA to stimulate 40 S subunit recruitment, an interaction observed in yeast, plants, and animals [7, 8]. An interaction between eIF4B and PABP, observed in plants and animals, serves to increase the RNA-binding affinity of PABP [9, 10, 11].

Two eIF4G isoforms are present in plants as in many eukaryotes [12]. However, unlike other eukaryotes, the plant eIF4G isoforms, referred to as eIF4G and eIFiso4G, differ substantially in size and sequence and each interacts with distinct eIF4E isoforms [13]. Nevertheless, both eIF4G isoforms from wheat interact with eIF4B, eIF4A, and PABP. Wheat eIF4G contains two interaction domains for these three partners whereas eIFiso4G has a single domain for eIF4B and PABP [14, 15]. Such divergence has suggested functional specialization in mRNA selection, a conclusion supported by the observation that alfalfa mosaic virus RNA 4 and *Arabidopsis* HSP21 use wheat eIF4G or eIFiso4G equally well whereas barley α-amylase, oat globulin mRNAs, and the tobacco etch virus 5′-leader use eIF4G preferentially [16, 17].

Translation from mRNAs containing the 5′-leader sequence from tobacco mosaic virus (TMV) (known as Ω) preferentially uses eIF4G over eIFisoG1 in wheat germ lysate [18]. More recent work revealed that in addition to eIF4G, Ω-mediated translation in *Arabidopsis* was also facilitated by the eIFiso4G2 isoform [19]. *Arabidopsis* expresses two eIFiso4G isoforms (eIFiso4G1 and eIFiso4G2) as do other species in the Brassicaceae and the Cleomaceae whereas species outside these species express only eIFiso4G1 isoforms [19]. eIFiso4G1 and eIFiso4G2 exhibit substantial sequence divergence, supporting their functional difference in facilitating Ω-mediated translation. The presence of eIFiso4G1 as the only eIFiso4G isoform in wheat explains the preferential use of eIF4G by Ω in wheat germ translation lysate [18].

PABP is highly conserved throughout eukaryotes and is involved in translation, mRNA biogenesis, mRNA transport, and mRNA stability [20]. Although its physical interaction with eIF4G serves to promote translation, PABP also increases mRNA stability by inhibiting DCP1/2-mediated decapping in yeast [21, 22]. Binding of eIF4E to the 5’-cap may be destabilized following loss of PABP during mRNA deadenylation, making the cap more susceptible to DCP1/2. Inhibition of decapping by PABP is also observed in mammals [23]. PABP promotes poly(A) nuclease (PAN)-mediated deadenylation but inhibits Caf1/Not1-mediated deadenylation in yeast [21, 22].

While yeast expresses just a single and essential PABP [24], PABP in *Arabidopsis* is expressed by a highly divergent, multigene family composed of eight members that can be grouped into three classes based on sequence similarity and gene structure [25]. Three members of the family, i.e., *PAB2*, *PAB4*, and *PAB8*, comprise class II PABP genes which are widely expressed, particularly in vegetative tissues [26, 27, 28, 29]. Expression of class I genes (*PAB3* and *PAB5*) is restricted to reproductive tissues whereas class III genes (*PAB6* and *PAB7*) exhibit weak and restricted expression. *PAB1*, originally considered an “orphan gene” is actually a class I gene expressed weakly in roots, pollen, and during late embryo development [25, 30, 31].

Analysis of the evolution of the PABP gene family in plants revealed that PABP expanded from a single gene in marine algae to two genes in fresh water algae which underwent further expansion to four highly similar progenitor genes during the evolution of non-vascular plants. These progenitor genes are most structurally similar to class I reproductive members of the *Arabidopsis* PABP gene family but all three classes of PABP genes present in higher plants evolved from these progenitor genes [25]. By the appearance of *Selaginella moellendorffii*, an early vascular plant, the first class III gene (i.e., *PAB7*) had appeared and a class II family member appears first in gymnosperms such as pine [25]. The three class II family members of *Arabidopsis* arose from a single class II gene in *Amborella trichopoda*, the most recent common ancestor of all extant flowering plants. However, *PAB8* appears largely only in the Brassicaceae and its sequence similarity suggests that it is a recent duplication of *PAB2* [25]. Similarly, *PAB3* is likely a duplication of *PAB5* that is present largely in the Brassicaceae and *PAB1* also appears limited to the Brassicaceae, demonstrating a recent expansion of this gene family in these species [25]. Finally, *PAB6* is present in rosids but not in asterid species.

The presence of a large gene family containing divergent members, suggests possible functional specialization that is supported by studies of the ability of members of the *Arabidopsis* gene family to compliment various PABP functions in *pab1* mutant yeast. Although expression of *Arabidopsis* PAB2, PAB3, and PAB5 in *pab1* yeast rescued viability, PAB2 and PAB5 promoted translation initiation and poly(A) shortening in yeast; PAB2, but not PAB5, protected the mRNA 5’-cap against decapping; and PAB2 interacted with eIF4G [27, 28, 32]. The function of other PABP family members, such as PAB1, PAB4, PAB6, and PAB7, has not been examined.

Given the importance of the interaction between eIF4G and PABP and its conserved nature throughout eukaryotes, how the isoforms of eIF4G and PABP in plants interact functionally to control specific aspects of plant growth and development is an important question that has remained unanswered. In this study, the functional interactions among eIF4G isoforms and those members of the PABP gene family that are widely-expressed were examined. Loss of PAB2 or PAB8 expression in combination with loss of eIF4G or eIFiso4G expression had little to no effect on growth or fertility whereas *pab2 pab8 eif4g* triple mutants or *pab2 pab8 eifiso4g1/2* quadruple mutants exhibited smaller stature than did *eif4g* or *eifiso4g1/2*, respectively. *pab4 eifiso4g1* mutant plants grew normally and were fertile but *pab4 eif4g* or *pab4 eifiso4g2* mutants could not be isolated. Moreover, *pab4/PAB4 eif4g/eIF4G* heterozygous plants exhibited low fertility. Analysis of mutant co-inheritance in reciprocal crosses with wild-type plants indicated that most ovaries and pollen from *pab4/PAB4 eif4g/eIF4G* plants were *PAB4 eif4g*. Similar results were obtained for co-inheritance studies with *pab4/PAB4 eifiso4g2/eIFiso4G2* plants. Co-inheritance of *pab4 eif4g* or *pab4 eifiso4g2* through the female germ line was observed in crosses with wild-type plants, albeit at low frequencies, demonstrating that the functional interaction between PAB4 and eIF4G or between PAB4 and eIFiso4G2 was not absolutely essential for ovary development. In contrast, co-inheritance of *pab4 eif4g* through the male germ line was not observed although inheritance of *pab4 eifiso4g2* was at least under conditions of eIFiso4G1 expression. These results suggest that the functional interactions between PAB4 and eIF4G and between PAB4 and eIFiso4G2 are essential for aspects of plant development including embryogenesis and/or vegetative growth.

## Materials and methods

### Plant growth

*Arabidopsis* seeds were imbibed and cold-treated for four days prior to germination in soil. Plants were grown in a growth room supplemented with Sylvania Gro-Lite fluorescent bulbs (Sylvania, Danvers, MA) at a photon flux density (PFD) of 100 μmol photons m^−2^ s^−1^.

### PCR analysis

DNA was isolated by quick-freezing plant material in liquid nitrogen, grounding to a fine powder, and resuspending 100 mg in 400 μl extraction buffer (100 mM Tris-Cl pH 9.0, 20 mM EDTA, 200 mM NaCl, 1% Sarcosyl, and 1% β-ME). Following centrifugation, the supernatant was extracted with 400 μl phenol: chloroform (1:1) and centrifuged to separate the phases. The DNA was precipitated from the aqueous phase by sodium acetate and isopropyl alcohol, washed with 75% ethanol and resuspended in H_2_O. PCR amplification was performed in 20 μl reactions containing 1 x PCR buffer, 0.4 u Taq DNA polymerase (Bioneer Inc Alameda, CA), 250 μM dNTPs, 10 μM forward and reverse primers, and 50 ng genomic DNA. Reactions were carried out using the following conditions: 95°C/5 min (1 cycle); 95°C/30 sec, 55°C/30 sec, 72°C/1 min (35 cycles); and a final extension at 72°C/5 min (1 cycle). To detect the presence of eIF4G, a forward primer, 080031LT (5′-TTCAGGTGCGAAGGAGAATGC-3′), and a reverse primer, 080031RP (5′-TGATGGCCCTTGTAGTACTTGCC-3′), were used and for *eif4g*, 080031LT and LBb1 (5’-GCGTGGACCGCTTGCTGCAACT-3’) were used. To detect the presence of eIFiso4G1, a forward primer, LF009905 (5′-TCATCACATTGTTCAGGTTAACACC-3′), and a reverse primer, RT009905 (5′- TTCGCTCAACTTGGGACCACT-3′), were used and for *eifiso4g1*, LF009905 and LBb1 were used. To detect the presence of eIFiso4G2, a forward primer, 076633LF (5′-TCAACCTTCAAACACAAAAGCTGA-3′), and a reverse primer, 076633RT (5′-AACCCTTTTCCCCGTCAAGGT-3′), were used and for *eifiso4g2*, 076633LF and LBb1 were used. To detect the presence of PAB2, a forward primer, PAB2-LP (5′-AACGGCTGAGATCAATCTCAC-3′), and a reverse primer, PAB2-RP (5′-GCGCTGGCAACATTTTTATTA-3′), were used and for *pab2*, PAB2-RP and LBb1.3 (5′- ATTTTGCCGATTTCGGAAC-3′) were used. To detect the presence of PAB4, a forward primer, PAB4-LP (5′-TGGGGTTGGGAATTTGTTTGT-3′), and a reverse primer, PAB4-RP (5′-GCCTTCTCTGCTGCCAGTGAA-3′), were used and for *pab4*, PAB4-RP and LBb1.3 were used. To detect the presence of PAB8, a forward primer, PAB8-LP (5′-TCATGCCTATGATGCAACAAG-3′), and a reverse primer, PAB8-RP (5′-GGTTTAAAAGCTTCGAATGATCA-3′), were used and for *pab8*, PAB8-RP and LBb1.3 were used.

### Chlorophyll measurements

Chlorophyll a and b were measured spectrophotometrically. Leaf samples were ground in liquid nitrogen and extracted with 90% (v/v) acetone. The absorbance at 664 and 647 nm was determined and used to calculated chlorophyll a and b content by the equations: Chl a = 11.93A_**664**_-1.93A_**647**_ and Chl b = 20.36A_**647**_-5.50A_**664**_, respectively. Measurements were repeated 2–3 times and representative results presented.

## Results

### The functional interaction between *PAB4* and *eIF4G* is necessary for fertility and vegetative growth

Class II members of the PABP gene family (*PAB2*, *PAB4*, and *PAB4*) are widely expressed and predominate in vegetative tissues [26, 27, 28, 29]. T-DNA insertion mutants of these gene members result in loss of expression but loss of any one class II member has little to no effect on growth or fertility [33]. Although the *pab4 pab8* double mutant also grows similar to wild-type (WT) plants, the *pab2 pab8* double mutant is slightly more compact than wild-type as a result of a shorter petiole (i.e., leaf stem) and reduced internode length best seen in the inflorescence (i.e., flower head) in the distance between siliques (i.e., seed pods) and the leaves of the *pab2 pab4* double mutant are substantially smaller and fewer in number than WT [33]. However, loss of any one eIF4G isoform (eIF4G, eIFiso4G1, or eIFiso4G2) fails to affect growth or fertility [34]. To examine whether the loss of expression of any one class II PABP gene member in combination with eIF4G would affect growth not observed for the individual mutants, single and double *pab* mutant plants were crossed with *eif4g* plants to generate each combinatorial mutant.

**Fig 1:**
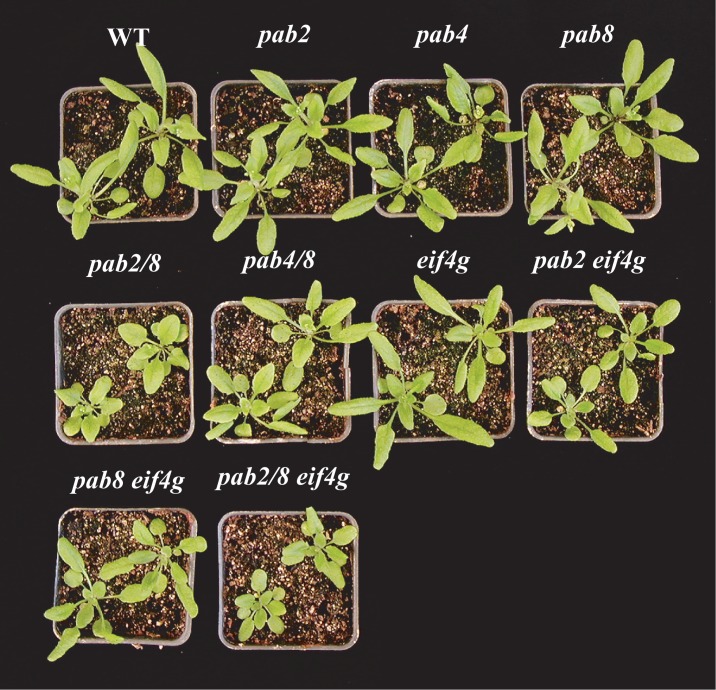
Growth characteristics of *pab2*, *pab8*, and *eif4g* combinatorial mutants. Adult wild-type and mutant plants at the first appearance of the inflorescence.

**Fig 2:**
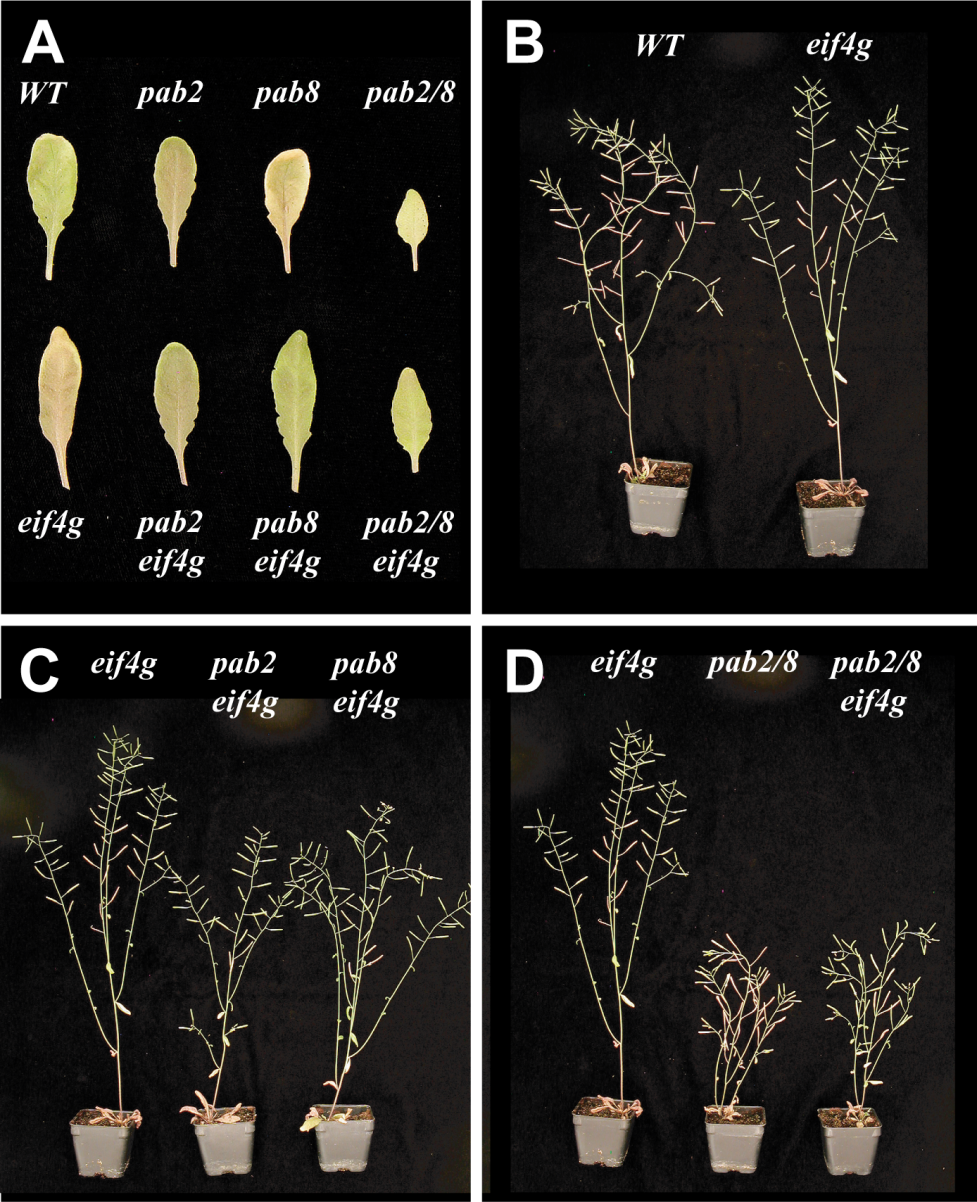
The effect of *pab2*, *pab8*, and *eif4g* combinatorial mutations on leaf and inflorescence growth. (A) Leaves from young adult wild-type and mutant plants. (B-D) Flowering wild-type and mutant plants with the inflorescence displayed.

The combination of *pab2* or *pab8* with *eif4g* had little effect on plant stature (Fig 1) or on leaf size of young adult plants (Fig 2A). However, the inflorescence of each was slightly reduced in size relative to the single mutants or to WT plants (Fig 2C). Combining the *pab2 pab8* double mutant with *eif4g* resulted in a more compact stature compared to the *eif4g* mutant but was similar to that observed for the *pab2 pab8* double mutant prior to flowering (Figs 1 and 2A) and a more compact inflorescence due to a shorter internode distance similar to that observed for the *pab2 pab8* double mutant (Fig 2D). The *pab2 eif4g* double mutant and *pab2 pab8 eif4g* triple mutant developed fewer ovaries per silique and shorter siliques than WT or any single mutant of these loci (Table 1) [33].

**Table 1:** Effect of combinatorial loss of class II PABP members and eIF4G on seed development.

	Ovaries/silique	% seed set	Siliques/plant	% silique set	Silique length
**WT**	49.4 ± 1.43	98.9	52.0 ± 4.55	99.1	14.4 ± 0.63
***eif4g***	49.9 ± 2.29	95.7***	44.5 ± 3.11	99.3	14.3 ± 0.60
***pab2 eif4g***	40.1 ± 3.28***	96.8*	54.7 ± 2.1	100	12.2 ± 1.00***
***pab8 eif4g***	47.1 ± 2.45*	97.3*	52.0 ± 4.00	99.3	13.8 ± 0.81*
***pab2/8 eif4g***	37.5 ± 5.29***	94.6***	41.3 ± 6.11	97.7	11.6 ± 1.29***
***pab2/8***	41.2 ± 2.93***	95.8***	52.0 ± 4.58	98.7	12.3 ± 1.24***

*, P < 0.05

**, P < 0.01

***, P < 0.001

**Fig 3:**
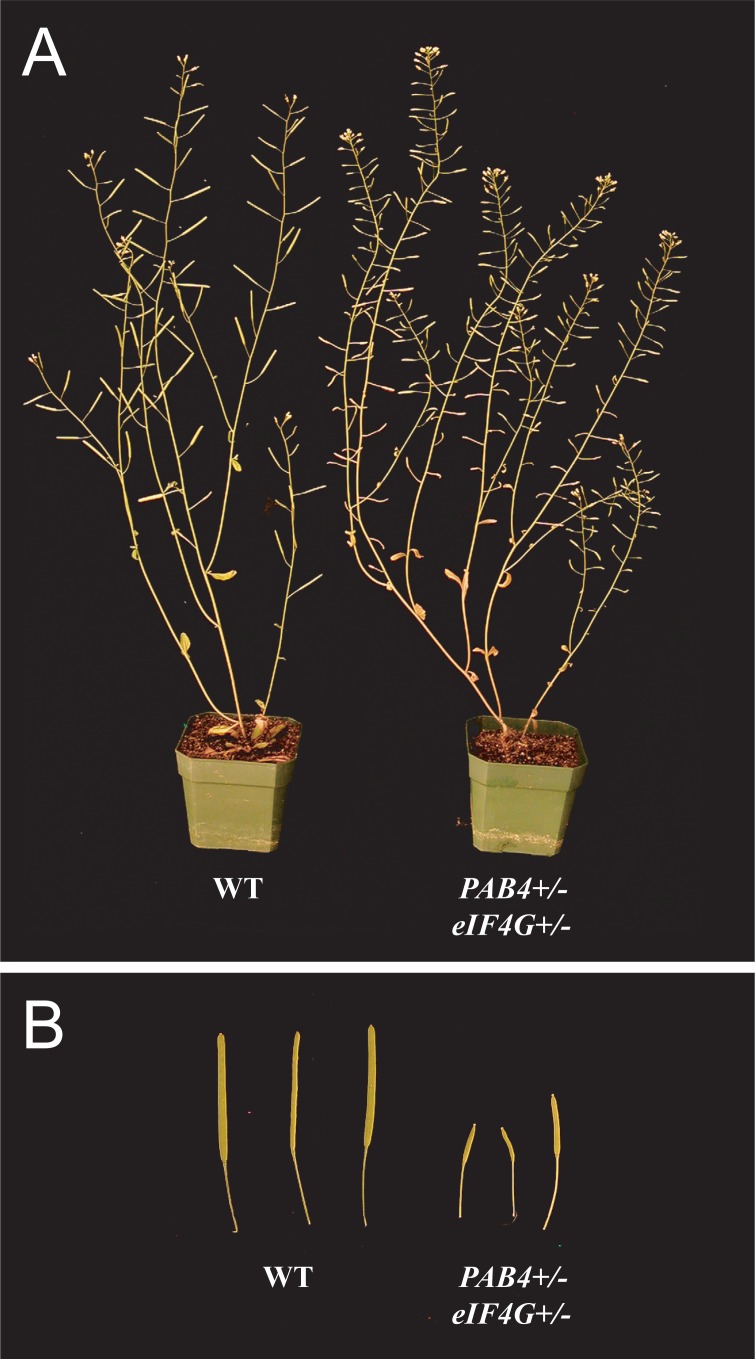
Development of the inflorescence and siliques in *pab4/PAB4 eif4g/eIF4G* heterozygous plants. (A) Inflorescence and (B) siliques of wild-type and *pab4/PAB4 eif4g/eIF4G* plants.

**Fig 4:**
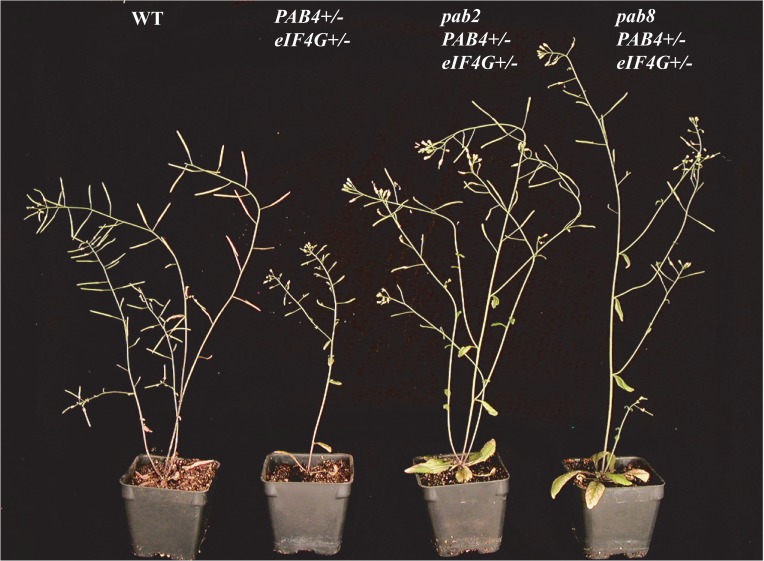
The effect of *pab2* or *pab8* on the growth of *pab4/PAB4 eif4g/eIF4G* heterozygous plants. Flowering wild-type and mutant plants with the inflorescence displayed.

Despite at least 15 attempts, isolation of a *pab4 eif4g* double mutant failed suggesting that loss of PAB4 and eIF4G results in synthetic lethality. Similarly, *pab2 pab4 eif4g* or *pab4 pab8 eif4g* triple mutants could not be isolated, supporting the conclusion that the combination of PAB4 and eIF4G is essential. Even attempts to isolate *pab4/PAB4 eif4g*/*eif4g* or *pab4*/*pab4 eif4g/eIF4G* mutants were unsuccessful. Isolation of a *pab4/PAB4 eif4g/eIF4G* double heterozygous mutant, however, was possible. *pab4/PAB4 eif4g/eIF4G* plants grew relatively normally although they exhibited reduced chlorophyll content (Table 2). *pab4/PAB4 eif4g/eIF4G* flowered normally (S1 Fig), however the plants exhibited extremely poor fertility with fewer ovaries per silique than WT (Fig 3 and Table 3). The failure of most ovaries to develop into embryos could be seen during embryogenesis (S1 Fig). The plants continued to flower for longer than WT, resulting in a substantial increase in silique number although most siliques were devoid of seed (Fig 3). Those siliques that did contain seed were much reduced in size, likely as a result of the presence of a single or few seed (Table 3 and S1 Fig). Although the fertility of *pab4/PAB4* heterozygote plants was unaffected (Table 3 and S2 Fig), *eif4g/eIF4G* heterozygote plants exhibited fewer ovaries per silique as in *pab4/PAB4 eif4g/eIF4G* plants (Table 3 and S3 Fig). However, the seed set per silique in *eif4g/eIF4G* heterozygote plants was more than three times higher than in *pab4/PAB4 eif4g/eIF4G* plants and, as a consequence, the siliques in *eif4g/eIF4G* heterozygote plants were longer to accommodate the increased number of seed present. Interestingly, despite *pab4/PAB4 eif4g/eIF4G* plants having far more siliques per plant relative to *eif4g/eIF4G* heterozygote plants, the extremely low seed set in the former resulted in fewer seed. Thus, as the reduction in fertility in *pab4/PAB4 eif4g/eIF4G* was greater than that observed in *eif4g/eIF4G* heterozygote plants, the addition of *PAB4* in a heterozygous state contributed to the reduction in fertility associated with the *eif4g/eIF4G* heterozygous mutation. Ovary and seed development in *pab2/PAB2 eif4g/eIF4G* and *pab8/PAB8 eif4g/eIF4G* plants was similar or slightly more impaired than that observed in *eif4g/eIF4G* heterozygote plants (Table 3 and S1 Fig). As *pab2 eif4g* and *pab8 eif4g* double homozygotes were viable whereas *pab4 eif4g* could not be isolated, these results suggest that the combination of *PAB4* and *eIF4G* is essential for plant viability and/or fertility. Although neither *PAB2* or *PAB8* was essential for plant viability and fertility, loss of expression of either partially ameliorated the growth defects and reduction in fertility observed in *pab4/PAB4 eif4g/eIF4G* plants through an increase in ovaries per silique and siliques with seed to a level similar to that observed for *eif4g/eIF4G* heterozygote plants (Fig 4 and Table 3), suggesting possible genetic interactions between *PAB4* and either *PAB2* or *PAB8*. An analysis of variance (one way ANOVA) with post hoc comparisons using the Tukey HSD test supported those differences that were determined significant by t-test (Table 3, S1 Table, S2 Table, and S3 Table).

**Table 2:** Chlorophyll content in adult leaves of WT and *pab4/PAB4 eif4g/eIF4G* plants.

	Chl a		Chl b		
Line	Chl a/mg FW	SD	Chl b/mg FW	SD	Chl a/Chl b
**WT**	0.920	0.069	0.252	0.030	3.67
***pab4/PAB4 eif4g/eIF4G***	0.665	0.029	0.175	0.002	3.81

**Table 3:** The combinatorial requirement of *PAB4* and *eIF4G* for fertility.

	Ovaries/silique	% seed set	Siliques/plant	% silique set	Silique length
**WT**	49.4 ± 1.43	98.0	52.0 ± 4.55	99.1	14.4 ± 0.63
***pab2*+/-**	31.8 ± 4.69***	63.4***	57.3 ± 8.74	100	11.4 ± 0.63***
***pab4*+/-**	46.7 ± 6.33	99.6	53.7 ± 5.69	99.4	15.1 ± 1.10*
***pab8*+/-**	41.5 ± 3.67***	96.2**	53.7 ± 5.86	100	14.0 ± 1.47
***eif4g*+/-**	31.6 ± 4.88***	72.0***	49.7 ± 3.06	96.0	12.9 ± 0.95***
***pab2*+/- *eif4g*+/-**	23.7 ± 4.31***	64.7***	61.3 ± 2.52	95.1*	11.9 ± 0.90***
***pab4*+/- *eif4g*+/-**	33.3 ± 6.58***	21.9***	261 ± 55.0***	33.4***	5.84 ± 1.07***
***pab8*+/- *eif4g*+/-**	21.1 ± 3.40***	58.4***	60.7 ± 9.50	94.3*	12.2 ± 0.69***
***pab2*+/- *pab8*+/-*eif4g*+/-**	34.0 ± 3.07***	64.1***	54.3 ± 6.03	100	12.5 ± 1.06***
***pab2 pab4*+/- *eif4g*+/-**	37.7 ± 5.53***	75.6***	94.6 ± 14.81***	95.1	14.0 ± 1.24
***pab8 pab4*+/- *eif4g*+/-**	32.7 ± 2.98***	78.8***	86.6 ± 8.65***	97.8	13.9 ± 0.70

*, P < 0.05

**, P < 0.01

***, P < 0.001

### The functional interaction between *PAB4* and *eIF4G* is required for seed development

The inability to isolate a *pab4 eif4g* double mutant and the poor fertility of *pab4/PAB4 eif4g/eIF4G* heterozygote plants suggested the possibility of altered Mendelian inheritance of the mutant loci in progeny following self-pollination. Genotypic analysis of such progeny revealed 54 out of 80 plants (67.5% versus expected of 6.25%) were *PAB4/PAB4 eif4g/eif4g* with 19 out of 80 (23.8% versus expected of 12.5%) being *PAB4/PAB4 eif4g/eIF4G* (Table 4). Four (5% versus expected of 6.25%) were *pab4/pab4 eIF4G/eIF4G* and 3 (3.75% versus expected of 25.0%) were *pab4/PAB4 eif4g/eIF4G* heterozygotes. These results deviate substantially from the expected inherence from a double heterozygous parent. All seed were viable suggesting that the combinatorial loss of *PAB4* and *eIF4G* expression affected germ line development or embryogenesis, consistent with the poor seed set exhibited by the heterozygous parent and the absence of double mutants among the progeny. To investigate whether loss of *PAB4* and *eIF4G* expression affected germ line development, *pab4/PAB4 eif4g/eIF4G* plants were crossed reciprocally with WT plants to examine co-inheritance distributions in ovaries and pollen that resulted in successful fertilization. Genotypic analysis of progeny from crosses using *pab4/PAB4 eif4g/eIF4G* plants as the recipient of WT pollen indicated that almost all (93.0%) ovaries resulting in successful fertilization events were *PAB4 eif4g* with only 2.3% being *pab4 eIF4G* and 4.7% being *pab4 eif4g* (Table 5). Interestingly, no *PAB4 eIF4G* ovaries were detected. Genotypic analysis of progeny from crosses using *pab4/PAB4 eif4g/eIF4G* plants as the pollen donor onto WT plants indicated that almost all (93.6%) pollen resulting in successful fertilization events were *PAB4 eif4g* with 2.1% being *pab4 eIF4G* and 4.3% being *PAB4 eIF4G* (Table 5). The predominance of the *PAB4 eif4g* combination in ovaries and pollen of *pab4/PAB4 eif4g/eIF4G* plants is consistent with the predominance of *PAB4/PAB4 eif4g/eif4g* in progeny of self-pollinated *pab4/PAB4 eif4g/eIF4G* heterozygote plants (Table 4). The detection of two *pab4 eif4g* ovaries in crosses with WT as the pollen donor (Table 5) demonstrates that expression of PAB4 and eIF4G is not essential for female gametophyte development although *pab4 eif4g* ovaries involved in successful fertilization events were under represented. No evidence supporting the presence of *pab4 eif4g* pollen was observed. This and the failure to isolate *pab4 eif4g* plants suggests synthetic lethality between *pab4* and *eif4g* during male or female gametophyte development/function and/or subsequent stages during embryo development in ways that the combination of *pab2* and *eif4g* or *pab8* and *eif4g* is not. Examination of pollen viability [35] as described (S1 Text) in flowers of *pab4/PAB4 eif4g/eIF4G* plants indicated that those pollen that developed to maturity were largely viable (S4 Fig).

**Table 4:** *pab4/PAB4 eif4g*/*eIF4G* heterozygous plants exhibit non-Mendelian inheritance.

	Inheritance rate								
	*PAB4/PAB4 eIF4G/eIF4G*	*PAB4/pab4 eIF4G/eIF4G*	*pab4/pab4 eIF4G/eIF4G*	*PAB4/PAB4 eIF4G/eif4g*	*PAB4/pab4 eIF4G/eif4g*	*pab4/pab4 eIF4G/eif4g*	*PAB4/PAB4 eif4g/eif4g*	*PAB4/pab4 eif4g/eif4g*	*pab4/pab4 eif4g/eif4g*
**Observed**	0/80 (0%)	0/80 (0%)	4/80 (5%)	19/80 (23.8%)	3/80 (3.75%)	0/80 (0%)	54/80 (67.5%)	0/80 (0%)	0/80 (0%)
**Expected**	5/80 (6.25%)	10/80 (12.5%)	5/80 (6.25%)	10/80 (12.5%)	20/80 (25%)	10/80 (12.5%)	5/80 (6.25%)	10/80 (12.5%)	5/80 (6.25%)

**Table 5:** *PAB4* and *eIF4G* are required for correct pollen and egg development.

		Inheritance rate							
		Egg				Pollen			
Female	Male	*PAB4 eIF4G*	*pab4 eIF4G*	*PAB4 eif4g*	*pab4 eif4g*	*PAB4 eIF4G*	*pab4 eIF4G*	*PAB4 eif4g*	*pab4 eif4g*
***PAB4/pab4 eIF4G/eif4g***	**WT**	0/43 (0%)	1/43 (2.3%)	40/43 (93.0%)	2/43 (4.7%)				
**WT**	***PAB4/pab4 eIF4G/eif4g***					2/47 (4.3%)	1/47 (2.1%)	44/47 (93.6%)	0/47 (0%)
***pab2/pab2*** ***PAB4/pab4 eIF4G/eif4g***	**WT**	0/59 (0%)	30/59 (50.8%)	27/59 (45.8%)	2/59 (3.4%)				
**WT**	***pab2/pab2*** ***PAB4/pab4 eIF4G/eif4g***					0/56 (0%)	28/56 (50.0%)	28/56 (50.0%)	0/56 (0%)
***pab8/pab8*** ***PAB4/pab4 eIF4G/eif4g***	**WT**	0/42 (0%)	26/42 (61.9%)	15/42 (35.7%)	1/42 (2.4%)				
**WT**	***pab8/pab8*** ***PAB4/pab4 eIF4G/eif4g***					0/58 (0%)	32/58 (55.2%)	25/58 (44.8%)	0/58 (0%)
**Expected**		25%	25%	25%	25%				
	**Expected**					25%	25%	25%	25%

The combinatorial loss of expression of either *PAB2* or *PAB8* with *pab4/PAB4 eif4g/eIF4G* substantially increased the number of *pab4 eIF4G* ovaries and pollen in reciprocal crosses with WT plants (Table 5). Thus, in the absence of PAB2 or PAB8 expression, *pab4 eIF4G* ovaries and pollen participate in successful fertilization events whereas they are largely absent from such events in the presence of PAB2 or PAB8 expression, suggesting possible genetic interactions between *PAB4* and either *PAB2* or *PAB8* during male and female gametophyte development or function. *pab2 pab4 eif4g* and *pab8 pab4 eif4g* ovaries resulting in successful fertilization events were observed but not *pab2 pab4 eif4g* or *pab8 pab4 eif4g* pollen. Together with the failure to detect *pab4 eif4g* pollen in crosses using *pab4/PAB4 eif4g/eIF4G* plants (Table 5), this represents a total of 161 progeny from these three crosses in which co-inheritance of *pab4* and *eif4g* through the male germ line failed. The lack of *pab4 eif4g* pollen in any of these three crosses would prevent the generation of *pab4/pab4 eif4g/eif4g* embryos, regardless of whether such embryos would be viable or not.

The aberrant inheritance exhibited by *pab4/PAB4 eif4g/eIF4G* plants was compared to that of *pab4/PAB4* and *eif4g/eIF4G* plants. Progeny from self-pollinated *pab4/PAB4* plants exhibited near normal Mendelian inheritance (Table 6) in contrast to the predominance of *PAB4*/*PAB4* progeny in self-pollinated *pab4/PAB4 eif4g/eIF4G* plants (Table 4). The combination of loss of expression of either *PAB2* or *PAB8* with *pab4/PAB4* did not substantially affect inheritance of *pab4* and *PAB4* (Table 6). Progeny from self-pollinated *eif4g/eIF4G* plants exhibited non-Mendelian distribution in which 74.1% of the progeny were *eIF4G/eIF4G*, 17.2% were *eif4g/eIF4G*, and only 8.6% were *eif4g/eif4g* (Table 6) versus the expected 25%, 50%, and 25%, respectively. These results differ substantially from the predominance of *eif4g*/*eif4g* progeny in self-pollinated *pab4/PAB4 eif4g/eIF4G* plants (Table 4).

**Table 6:** Inheritance rate in *PAB4* and *eIF4G* heterozygotes.

Selfed Heterozygote	Inheritance rate		
	***PAB4/PAB4***	***PAB4/pab4***	***pab4/pab4***
***PAB4/pab4***	21/71 (29.6%)	37/71 (52.1%)	13/71 (18.3%)
***pab2/pab2 PAB4/pab4***	20/58 (34.5%)	30/58 (51.7%)	8/58 (13.8%)
***pab8/pab8 PAB4/pab4***	22/58 (37.9%)	33/58 (56.9%)	3/58 (5.2%)
	***eIF4G/eIF4G***	***eIF4G/eif4g***	***eif4g/eif4g***
***eIF4G/eif4g***	43/58 (74.1%)	10/58 (17.2%)	5/58 (8.6%)
**Expected**	25%	50%	25%

Reciprocal crosses between *pab4/PAB4* plants and WT demonstrated the inheritance rate of *PAB4* and *pab4* was approximately equal through the male and female germ lines (Table 7). Reciprocal crosses between *eif4g/eIF4G* plants and WT suggested the inheritance rate of *eIF4G* and *eif4g* was approximately equal through either germ line (Table 7).

**Table 7:** Inheritance of *pab4/PAB4* or *eif4g*/*eIF4G* heterozygous loci through the male or female germ line.

		Inheritance rate							
		Egg				Pollen			
Female	Male	*eIF4G*	*eif4g*	*PAB4*	*pab4*	*eIF4G*	*eif4g*	*PAB4*	*pab4*
***PAB4/pab4***	**WT**			30/58 (51.7%)	28/58 (48.3%)				
**WT**	***PAB4/pab4***							27/52 (51.9%)	25/52 (48.1%)
***eIF4G/eif4g***	**WT**	26/48 (54.2%)	22/48 (45.8%)						
**WT**	***eIF4G/eif4g***					18/40 (45.0%)	22/40 (55.0%)		

### The functional interaction between *PAB4* and *eIFiso4G2* is required for ovary and pollen function

The synthetic lethality between *pab4* and *eif4g* raised the question of whether a similar interaction exists between PAB4 and the other eIF4G isoform, eIFiso4G. eIFiso4G is encoded by two genes in *A*. *thaliana*: eIFiso4G1 (At5g57870) and eIFiso4G2 (At2g24050) which are substantially divergent (55.4% identity and 64.9% similarity). No visible phenotype is observed in *eifiso4g1* or *eifiso4g2* mutants other than the slightly reduced stature of *eifiso4g1* plants [19]. The eIFiso4G double null mutant, *i*.*e*. *eifiso4g1/2*, is considerably smaller with reduced chlorophyll levels and slower growth rate than WT plants [34, 36]. Although this suggests that the eIFiso4G isoforms are functionally similar in contributing to vegetative growth, they also exhibit functional differences in the mRNAs they support translationally [19].

**Fig 5:**
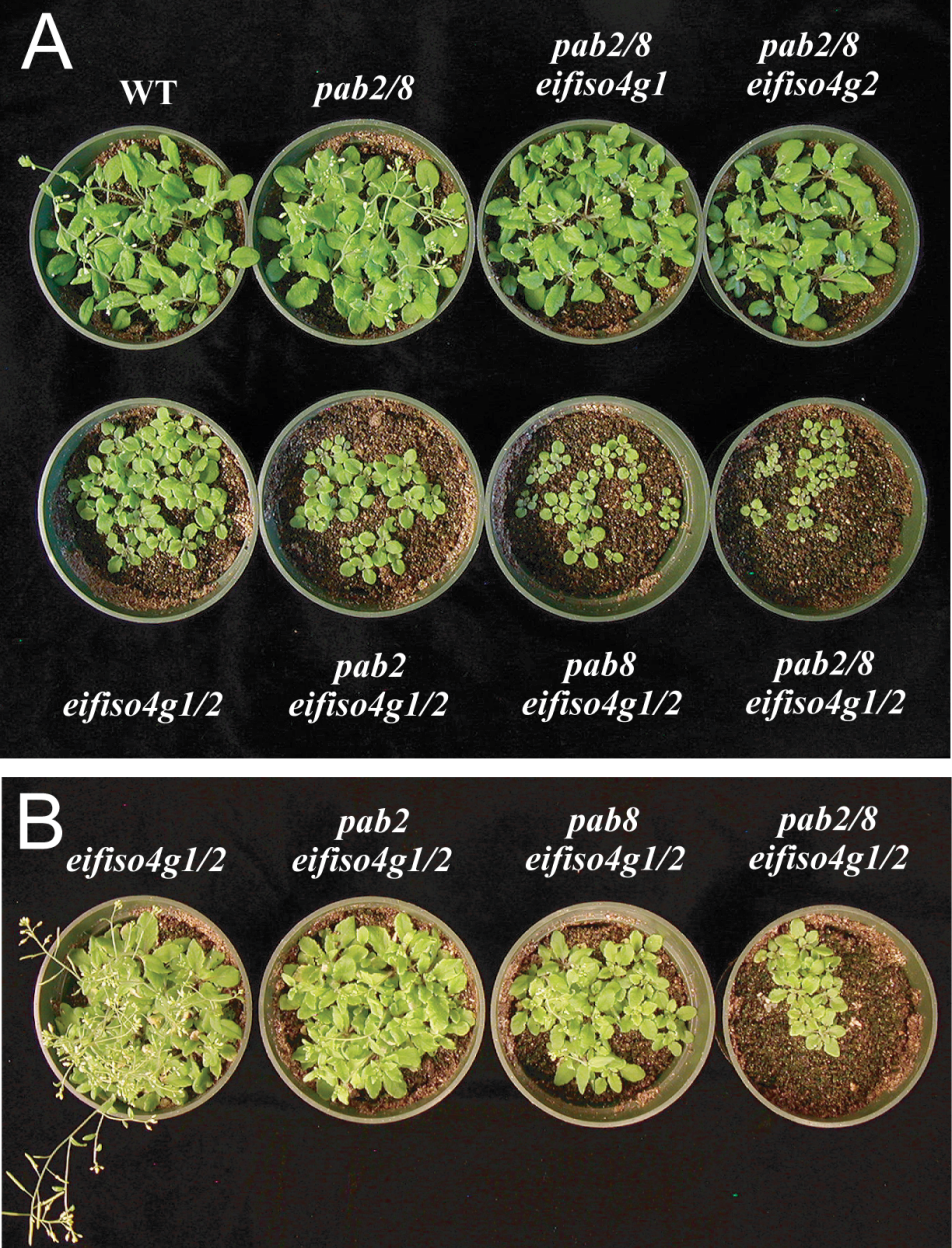
Growth characteristics of *pab2*, *pab8*, and *eifiso4g* combinatorial mutants. (A) Adult wild-type and mutant plants at the first appearance of the inflorescence in the wild-type. Mutants containing the *eifiso4g1/2* mutations are delayed in flowering. (B) Adult *eifiso4g1/2* mutant plants at flowering showing the effect of *pab2* and/or *pab8* mutations on growth and flowering time. The pots shown are the same as those in (A) but at flowering of the *eifiso4g1/2* mutant plants.

**Fig 6:**
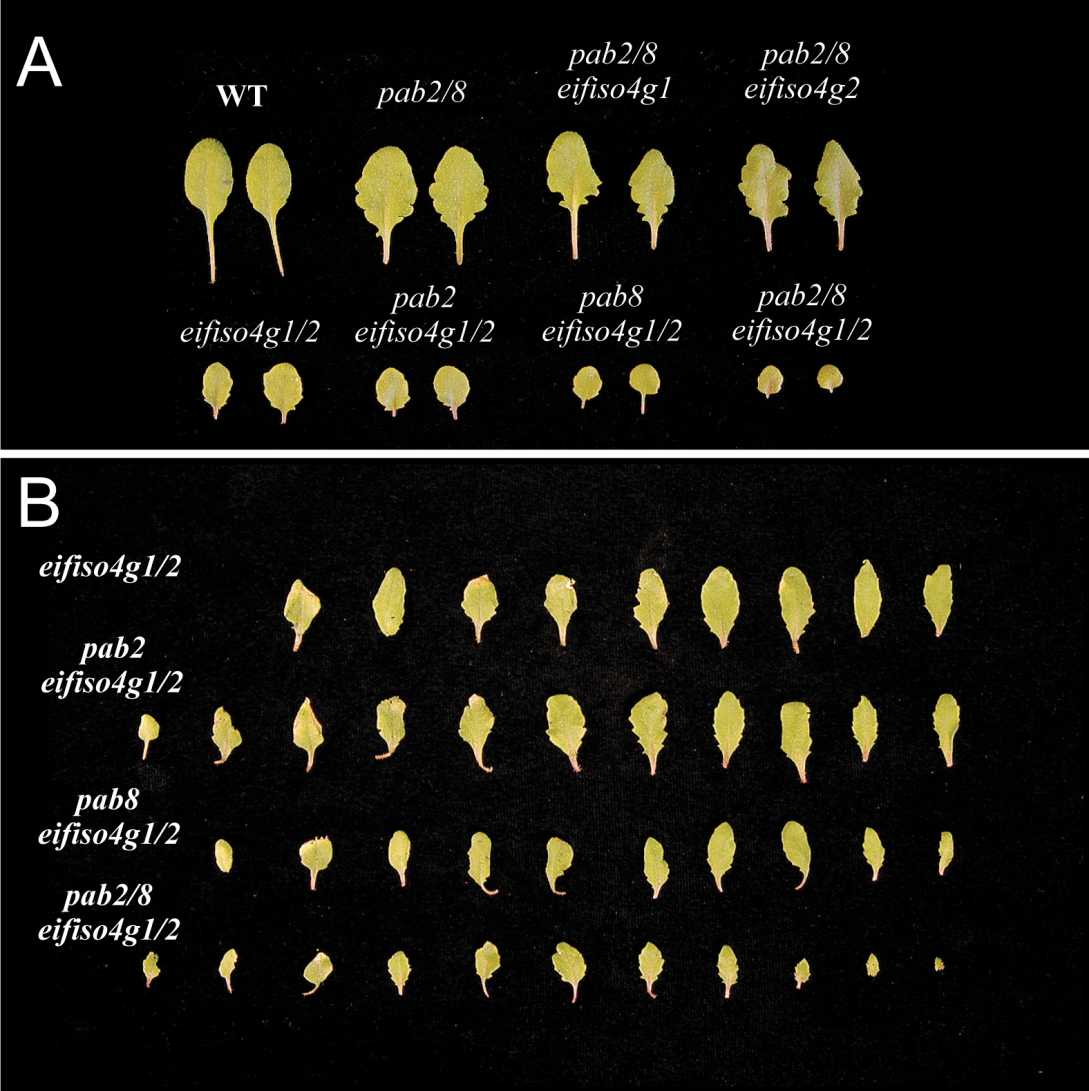
The effect of *pab2*, *pab8*, and *eifiso4g* combinatorial mutations on leaf size. (A) Adult leaves of fully flowering wild-type and mutant plants. (B) Leaf series from fully flowering adult *eifiso4g1/2* mutant plants showing the effect of *pab2* and/or *pab8* mutations on the size of every leaf from a representative plant.

**Fig 7:**
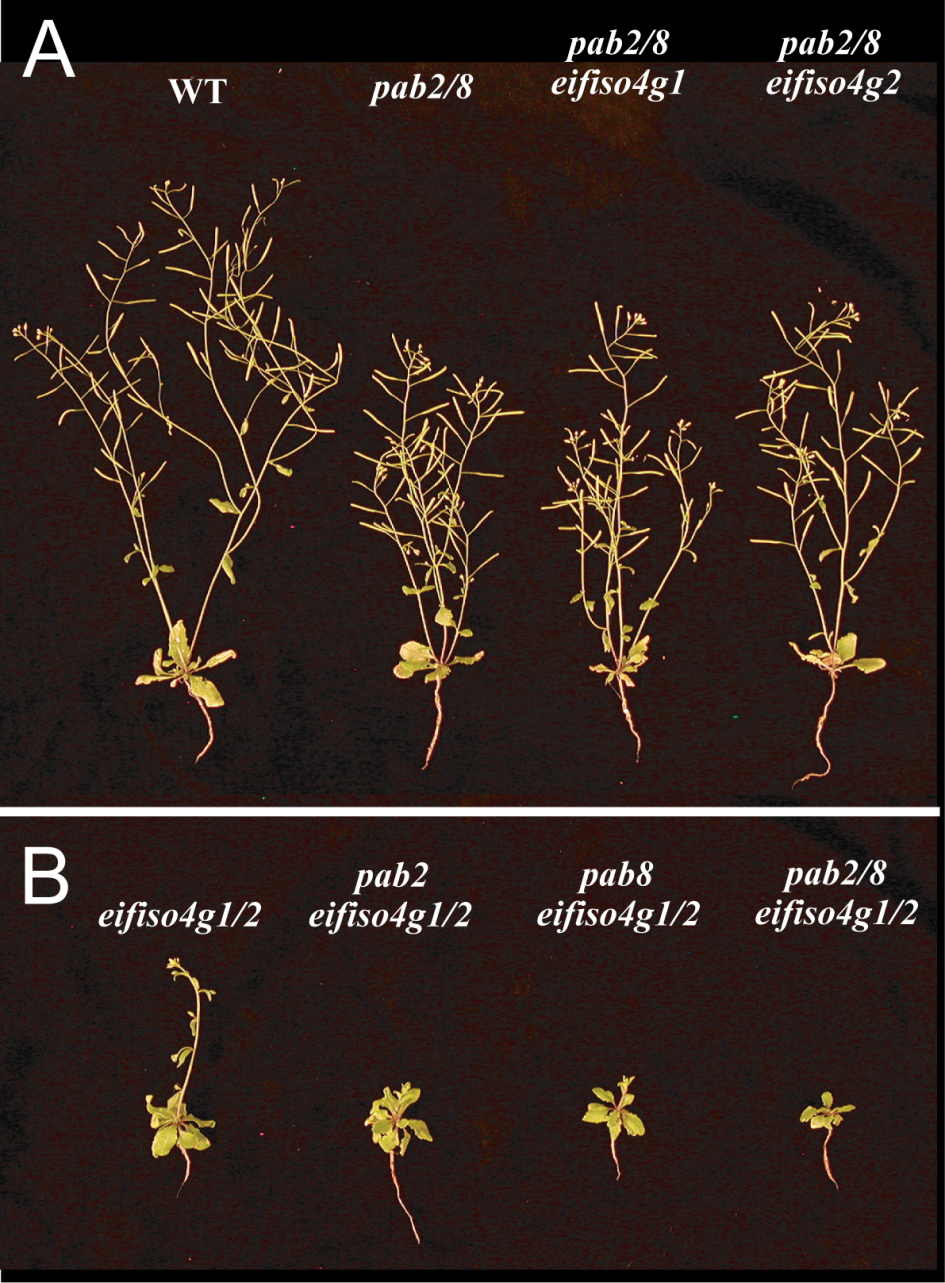
The effect of *pab2*, *pab8*, and *eifiso4g* combinatorial mutations on plant stature, flowering, and inflorescence size. (A) Adult wild-type and *eifiso4g* isoform mutants showing the effect of *pab2/8* mutations on plant and inflorescence growth. (B) Adult *eifiso4g1/2* mutant plants showing the effect of *pab2* and/or *pab8* mutations on plant stature and flowering time.

To examine whether the combinatorial loss of any of the widely expressed PABP gene members with eIFiso4G would affect growth, single and double *pab* mutant plants were crossed with *eifiso4g1* and *eifiso4g2* plants to generate each combination of mutants. *pab2 pab8 eifiso4g1* or *pab2 pab8 eifiso4g2* triple mutants were similar in stature to *pab2 pab8* double mutant and WT plants although these particular mutants are more compact than WT due to shorter petioles (Figs 5 and 6). The inflorescence of *pab2 pab8 eifiso4g1* or *pab2 pab8 eifiso4g2* triple mutants was also more compact than WT as it was in the *pab2 pab8* double mutant (Fig 7). The *eifiso4g1/2* double mutant is substantially reduced in stature and leaf size relative to WT plants or either single mutant (Figs 5 and 6). The growth rate of *pab2 eifiso4g1/2* and *pab8 eifiso4g1/2* triple mutants was slower than *eifiso4g1/2* plants and the leaf size of the *pab8 eifiso4g1/2* triple mutant was smaller than fully flowering *eifiso4g1/2* plants (Figs 5, 6 and 7). The growth rate of the *pab2 pab8 eifiso4g1/2* quadruple mutant was even slower with smaller stature and leaf size relative to either triple mutant (Figs 5, 6 and 7) and it exhibited delayed flowering even with respect to the *eifiso4g1/2* double mutant (Fig 7). These data suggest that *PAB2* and *PAB8* contribute combinatorially to plant stature which is observed whether *eIFiso4G* (or *eIF4G*) is expressed or not. These data also demonstrate that *pab2* and *pab8*, either together or alone, do not exhibit synthetic lethality with *eifiso4g*.

**Fig 8:**
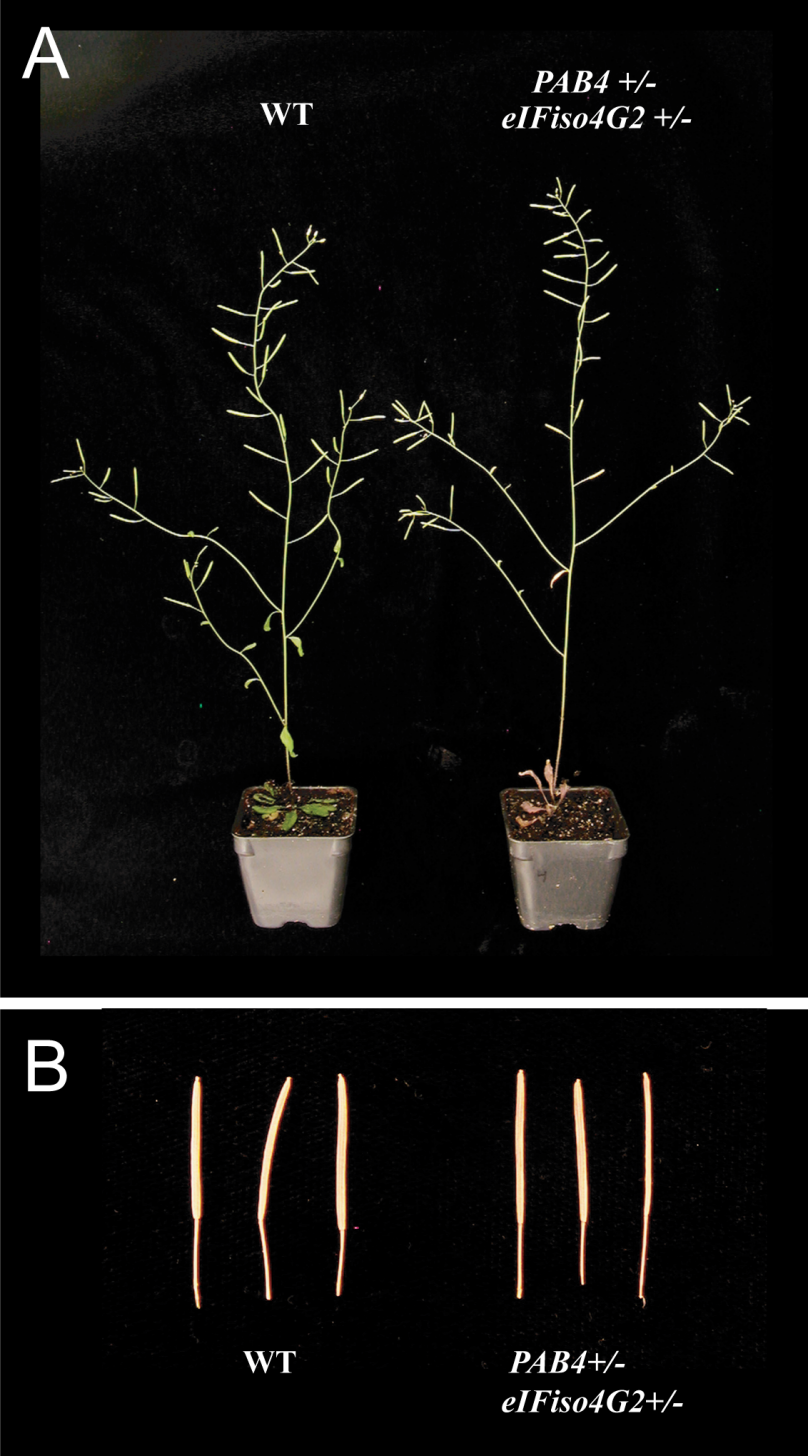
Development of the inflorescence and siliques in *pab4/PAB4 eifiso4g2/eIFiso4G2* heterozygous plants. (A) Inflorescence and (B) siliques of wild-type and *pab4/PAB4 eifiso4g2/eIFiso4G2* plants.

As with PAB4 and eIF4G, over 10 attempts to isolate a *pab4 eifiso4g1/2* triple mutant failed suggesting that loss of *PAB4* and *eIFiso4G* expression results in synthetic lethality. Similarly, *pab2 pab4 eifiso4g1/2* or *pab4 pab8 eifiso4g1/2* quadruple mutants could not be isolated, supporting the conclusion that the combination of *PAB4* and *eIFiso4G* is essential. As eIFiso4G is expressed as two divergent isoforms, the isolation of *pab4 eifiso4g1* or *pab4 eifiso4g2* double mutants was attempted. The *pab4 eifiso4g1* double mutant was viable, flowered normally, and exhibited fertility similar to WT (S5 Fig). The *pab4 eifiso4g2* double mutant, however, could not be isolated. Isolation of a *pab4/PAB4 eif4iso4g2/eIFiso4G2* double heterozygous mutant was possible. Its growth characteristics were similar to WT plants (Fig 8 and S5 Fig) and did not exhibit the reduced chlorophyll content (D. Gallie, personal observation) or poor fertility observed with *pab4/PAB4 eif4g/eIF4G* heterozygous plants. Interestingly, a cross between *pab4/pab4 eifiso4g1/eifiso4g1* and *eifiso4g1/eifiso4g1 eifiso4g2/eifiso4g2* plants to generate *pab4/PAB4 eifiso4g1/eifiso4g1 eif4iso4g2/eIFiso4G2* which were then selfed, resulted in a subset of progeny that consistently died following germination (S6 Fig). This suggests that *eIFiso4G1* expression was required to support growth in the partial or complete absence of *PAB4* and *eIFiso4G2* expression.

*pab4/PAB4 eif4iso4g2/eIFiso4G2* double heterozygous mutant plants exhibited similar fertility as WT or *pab2/PAB2 eif4iso4g2/eIFiso4G2*, *pab8/PAB8 eif4iso4g2/eIFiso4G2*, and *pab4/PAB4 eif4iso4g1/eIFiso4G1* plants (Table 8 and S5 Fig). In contrast, the *eif4iso4g1 eif4iso4g2* double mutant has substantially fewer ovaries per silique, reduced seed per silique, and fewer and shorter siliques. *eif4iso4g1/eIFiso4G1 eif4iso4g2/eIFiso4G2* double heterozygous mutant plants also had fewer ovaries per silique, reduced seed per silique, and slightly shorter siliques although not fewer siliques (Table 8). An analysis of variance (one way ANOVA) with post hoc comparisons using the Tukey HSD test supported those differences that were determined significant by t-test (Table 8, S4 Table, S5 Table, S6 Table, S7 Table, S8 Table, and S9 Table). These results indicate a synthetic lethal relationship between *PAB4* and *eIFiso4G2* similar to that observed between *PAB4* and *eIF4G*.

**Table 8:** Effect of combinatorial loss of class II PABP members and eIFiso4G on seed development.

	Ovaries/silique	% seed set	Siliques/plant	% silique set	Silique length
**WT**	49.4 ± 1.43	98.0	52.0 ± 4.55	99.1	14.4 ± 0.63
***eifiso4g1***	45.8 ± 4.30*	98.6	48.0 ± 3.61	100	13.7 ± 0.61
***pab4 eifiso4g1***	55.2 ± 4.21	98.4	47.3 ± 5.12	100	15.8 ± 0.86***
***eifiso4g2***	45.5 ± 4.79*	98.2	50.0 ± 4.35	98.6	14.2 ± 0.86
***pab2 eifiso4g2***	44.5 ± 4.12**	98.1	53.0 ± 7.0	97.8	14.3 ± 0.59
***pab8 eifiso4g2***	48.5 ± 5.36	97.9	47.7 ± 4.16	98.0	13.9 ± 0.93
***eifiso4g1/2***	21.4 ± 4.46***	67.0***	18.6 ± 1.14***	96.6	6.94 ± 0.54***
***pab2*+/- *eifiso4g2*+/-**	42.6 ± 5.17**	98.5	54.7 ± 2.52	100	14.0 ± 0.76
***pab4*+/- *eifiso4g2*+/-**	45.7 ± 2.81**	97.0	61.0 ± 1.00	100	13.9 ± 0.47
***pab8*+/- *eifiso4g2*+/-**	41.2 ± 5.24**	98.0	54.3 ± 4.73	100	13.4 ± 0.93*
***eifiso4g1*+/-**	29.9 ± 4.14***	71.7***	53.0 ± 4.36	100	11.6 ± 0.76***
***eifiso4g2*+/-**	42.0 ± 10.4*	94.4*	53.7 ± 4.73	100	13.1 ± 1.22**
***pab4*+/- *eifiso4g1*+/-**	48.3 ± 4.42	98.7	59.5 ± 12.3	100	14.1 ± 0.99
***eifiso4g1/2*+/-**	28.3 ± 3.50***	61.1***	58.3 ± 3.51	99.4	10.3 ± 1.13***

*, P < 0.05

**, P < 0.01

***, P < 0.001

Because of the observed non-Mendelian inheritance observed in *pab4/PAB4 eif4g/eIF4G* plants, genotypic analysis of progeny from self-pollinated *pab4/PAB4 eif4iso4g2/eIFiso4G2* plants was performed. 22 out of 73 progeny (30.1%) were *pab4/PAB4 eif4iso4g2/eIFiso4G2*, similar to the 25% expected for the double heterozygote (Table 9). *PAB4/PAB4 eifiso4g2/eifiso4g2* and *pab4/pab4 eIFiso4G2*/*eIFiso4G2* progeny were overrepresented at 26.0% and 19.2%, respectively, compared to the 6.25% expected for each. No *pab4/pab4 eifiso4g2/eifiso4g2* progeny was present among the progeny. However, no *PAB4/PAB4 eIFiso4G2*/*eIFiso4G2* progeny was present either, as observed in progeny from self-pollinated *pab4/PAB4 eif4g/eIF4G* plants (Table 4). *pab4/PAB4 eIFiso4G2*/*eIFiso4G2*, *PAB4/PAB4 eif4iso4g2/eIFiso4G2*, and *pab4/pab4 eif4iso4g2/eIFiso4G2* progeny were underrepresented at 1.36%, 5.48%, and 1.36% compared to the 12.5% expected for each. All seed were viable indicating that the absence of certain mutant combinations may result from germ cell/embryo lethality or in differences in pollen germination or growth rate of the pollen tube (germinating pollen).

**Table 9:** *pab4/PAB4 eifiso4g2*/*eIFiso4G2* heterozygous plants exhibit non-Mendelian inheritance.

	Inheritance rate								
	*PAB4/PAB4 eIFiso4G2/ eIFiso4G2*	*PAB4/pab4 eIFiso4G2/ eIFiso4G2*	*pab4/pab4 eIFiso4G2/ eIFiso4G2*	*PAB4/PAB4 eIFiso4G2/ eifiso4g2*	*PAB4/pab4 eIFiso4G2/ eifiso4g2*	*pab4/pab4 eIFiso4G2/ eifiso4g2*	*PAB4/PAB4 eifiso4g2/ eifiso4g2*	*PAB4/pab4 eifiso4g2/ eifiso4g2*	*pab4/pab4 eifiso4g2/ eifiso4g2*
**Observed**	0/73 (0%)	1/73 (1.36%)	19/73 (26.0%)	4/73 (5.48%)	22/73 (30.1%)	1/73 (1.36%)	14/73 (19.2%)	10/73 (13.7%)	0/73 (0%)
**Expected**	4.6/73 (6.25%)	9.1/73 (12.5%)	4.6/73 (6.25%)	9.1/73 (12.5%)	18.2/73 (25%)	9.1/73 (12.5%)	4.6/73 (6.25%)	9.1/73 (12.5%)	4.6/73 (6.25%)

To determine whether certain mutant combinations affect ovary or pollen development, *pab4/PAB4 eif4iso4g2/eIFiso4G2* plants were reciprocally crossed with WT plants to examine which gene combinations predominated in ovaries and pollen participating in successful fertilization events. Genotypic analysis of progeny from crosses using *pab4/PAB4 eif4iso4g2/eIFiso4G2* plants as the recipient of WT pollen indicated that 3.5% ovaries were *PAB4 eIFiso4G2*, 31.6% ovaries were *pab4 eIFiso4G2*, 61.4% ovaries were *PAB4 eifiso4g2*, and 3.5% ovaries were *pab4 eifiso4g2* (Table 10). Genotypic analysis of progeny from crosses using *pab4/PAB4 eifiso4g2/eIFiso4G2* plants as the pollen donor onto WT plants indicated that 51.6% of successful fertilization events resulted from *pab4 eIFiso4G2* pollen, 41.9% resulted from *PAB4 eifiso4g2* pollen, 6.5% resulted from *pab4 eifiso4g2* pollen, and none from *PAB4 eIFiso4G2* pollen (Table 10). The presence of *pab4 eifiso4g2* ovaries and pollen, although few in number, demonstrates that the combination of PAB4 and *eIFiso4G2* is not essential for male or female gametophyte development (although they are underrepresented in each germ line) and thus would not prevent the generation of *pab4/pab4 eifiso4g2/eifiso4g2* embryos. The failure to isolate *pab4 eifiso4g2* plants therefore suggests synthetic lethality between *pab4* and *eifiso4g2* during embryo development.

**Table 10:** *PAB4* and *eIFiso4G2* are required for correct pollen and egg development.

		Inheritance rate							
		Egg				Pollen			
Female	Male	*PAB4 eIFiso4G2*	*pab4 eIFiso4G2*	*PAB4 eifiso4g2*	*pab4 eifiso4g2*	*PAB4 eIFiso4G2*	*pab4 eIFiso4G2*	*PAB4 eifiso4g2*	*pab4 eifiso4g2*
***PAB4/pab4 eIFiso4G2/eifiso4g2***	**WT**	2/57 (3.5%)	18/57 (31.6%)	35/57 (61.4%)	2/57 (3.5%)				
**WT**	***PAB4/pab4 eIFiso4G2/eifiso4g2***					0/62 (0%)	32/62 (51.6%)	26/62 (41.9%)	4/62 (6.5%)
***pab2/pab2*** ***PAB4/pab4 eIFiso4G2/eifiso4g2***	**WT**	0/64 (0%)	34/64 (53.1%)	30/64 (46.9%)	0/64 (0%)				
**WT**	***pab2/pab2*** ***PAB4/pab4 eIFiso4G2/eifiso4g2***					7/85 (8.2%)	38/85 (44.7%)	34/85 (40.0%)	6/85 (7.1%)
***pab8/pab8*** ***PAB4/pab4 eIFiso4G2/eifiso4g2***	**WT**	5/58 (8.6%)	31/58 (53.4%)	22/58 (37.9%)	0/58 (0%)				
**WT**	***pab8/pab8*** ***PAB4/pab4 eIFiso4G2/eifiso4g2***					8/54 (14.8%)	20/54 (37.0%)	20/54 (37.0%)	6/54 (11.1%)
**Expected**		25%	25%	25%	25%				
	**Expected**					25%	25%	25%	25%

To determine whether the absence of PAB2 expression might alter inheritance of *pab4* or *eifiso4g2* in male and female gametophytes, reciprocal crosses between *pab2/pab2 pab4/PAB4 eifiso4g2/eIFiso4G2* and WT plants were conducted and genotypic analysis of the progeny performed. Similar to reciprocal crosses between *pab4/PAB4 eifiso4g2/eIFiso4G2* and WT plants, 53.1% ovaries were *pab4 eIFiso4G2*, 46.9% ovaries were *PAB4 eifiso4g2*, and no *PAB4 eIFiso4G2* or *pab4 eifiso4g2* ovaries were detected (Table 10). In crosses between *pab2/pab2 pab4/PAB4 eifiso4g2/eIFiso4G2* as the pollen donor onto WT plants, 8.2% of successful fertilization events resulted from *PAB4 eIFiso4G2* pollen, 44.7% successful fertilization events resulted from *pab4 eIFiso4G2* pollen, 40.0% resulted from *PAB4 eifiso4g2* pollen, and 7.1% resulted from *pab4 eifiso4g2* pollen (Table 10).

In crosses between *pab8/pab8 pab4/PAB4 eifiso4g2/eIFiso4G2* as the recipient of WT pollen, 8.6% of the ovaries were *PAB4 eIFiso4G2*, 53.4% were *pab4 eIFiso4G2*, and 37.9% were *PAB4 eifiso4g2*, with no *pab4 eifiso4g2* ovaries detected (Table 10). In crosses using pollen from *pab8/pab8 pab4/PAB4 eifiso4g2/eIFiso4G2* plants for WT recipients, 14.8% of successful fertilization events resulted from *PAB4 eIFiso4G2* pollen, 37.0% successful fertilization events resulted from either *pab4 eIFiso4G2* pollen or *PAB4 eifiso4g2* pollen, and 11.1% resulted from *pab4 eifiso4g2* pollen (Table 10). These results suggest loss of PAB2 or PAB8 expression does not grossly alter the distribution of mutant loci in male or female gametophyte.

Genotypic analysis of progeny from crosses using *pab4/PAB4 eifiso4g1/eIFiso4G1* plants as the recipient of WT pollen indicated that 27.8% ovaries were *PAB4 eIFiso4G1*, 27.8% ovaries were *pab4 eIFiso4G1*, 15.3% ovaries were *PAB4 eifiso4g1*, and 29.2% ovaries were *pab4 eifiso4g1* (Table 11). When pollen from *pab4/PAB4 eifiso4g1/eIFiso4G1* plants was used in crosses with WT plants, 6.3% of successful fertilization events resulted from *PAB4 eIFiso4G1* pollen, with 37.5% resulting from *pab4 eIFiso4G1* pollen, 25.0% resulting from *PAB4 eifiso4g1* pollen, and 31.3% resulting from *pab4 eifiso4g1* pollen (Table 11). The prevalence of *pab4 eifiso4g1* ovaries and pollen resulting in successful fertilization events and the lack of observable phenotype in *pab4 eifiso4g1* plants demonstrates that the combinatorial expression of PAB4 and eIFiso4G1 is not essential for male or female gametophyte development, embryo development, or for supporting vegetative growth.

**Table 11:** *PAB4* and *eIFiso4G1* are required for correct pollen and egg development.

		Inheritance rate							
		Egg				Pollen			
Female	Male	*PAB4 eIFiso4G1*	*pab4 eIFiso4G1*	*PAB4 eifiso4g1*	*pab4 eifiso4g1*	*PAB4 eIFiso4G1*	*pab4 eIFiso4G1*	*PAB4 eifiso4g1*	*pab4 eifiso4g1*
***PAB4/pab4 eIFiso4G1/eifiso4g1***	**WT**	20/72 (27.8%)	20/72 (27.8%)	11/72 (15.3%)	21/72 (29.2%)				
**WT**	***PAB4/pab4 eIFiso4G1/eifiso4g1***					4/64 (6.3%)	24/64 (37.5%)	16/64 (25.0%)	20/64 (31.3%)
**Expected**		25%	25%	25%	25%				
	**Expected**					25%	25%	25%	25%

## Discussion

In this study, *pab4* and *eif4g* exhibited synthetic lethality as did *pab4* and *eifiso4g2*. In contrast, no synthetic lethality was observed between *pab4* and *eifiso4g1* or between other *pab* and *eif4g* or *eifiso4g* mutants. The synthetic lethality exhibited between either *eif4g* or *eifiso4g2* with *pab4* suggests that eIF4G and eIFiso4G2 are functionally similar but that their combined contribution is necessary. Interestingly, these results are consistent with the previous finding that eIF4G and eIFiso4G2 exhibit similar translational preferences in that translation from mRNAs containing the TMV Ω 5’-leader sequence preferentially uses eIF4G and eIFiso4G2 over eIFisoG1 in *Arabidopsis* [19].

PABP and eIF4G isoforms are expressed from multigene families. The *PAB* gene family in *Arabidopsis* (and in species of the Brassicaceae) is unusual in the number and diversity of its isoforms. From a single gene in the marine algae *Chlamydomonas reinhardtii* to two genes in fresh water algae (*Klebsormidium flaccidum*) to four genes in *Physcomitrella patens*, the *PAB* gene family expanded with the colonization of land [25]. By the appearance of gymnosperms and the basal angiosperm, *Amborella trichopoda*, the *PAB* gene family had differentiated into the three classes that characterize PABP proteins in higher plants with further expansion of each class, especially within the Brassicaceae [25].

Of the three class II *PAB* genes in *Arabidopsis*, *PAB2* and *PAB4* contribute most to vegetative growth and normal vegetative-to-floral transition [33]. *PAB2* contributes significantly to inflorescence architecture, silique morphology, and fertility [33]. *PAB8* also contributed to silique and inflorescence development and to fertility as revealed in double class II *pab* mutants. The contribution that class II PABP proteins make to flowering and fertility demonstrated that, despite the specific expression from the class I *PAB3* and *PAB5* genes and the class III *PAB7* gene in reproductive tissues [33], class II *PAB* genes are also important for reproduction. Of the three class II PABP isoforms in *Arabidopsis*, PAB4 is less similar to PABP from other species such as wheat (56.7%/63.6% identity/similarity) than is PAB2 (62.8%/68.2% identity/similarity) or PAB8 (66.4%/70.5% identity/similarity) suggesting potential for greater functional divergence. Interestingly, in knockouts of individual class II PAB members in *Arabidopsis*, expression from luciferase constructs with or without the TMV Ω 5’-leader sequence was reduced to a greater degree in *pab4* mutants than in *pab2* or *pab8* mutants, suggesting that *PAB4* contributes more to reporter gene expression than does either *PAB2* or *PAB8* [33]. These observations support the conclusion that class II *PAB* genes exhibit some degree of functional specialization.

Although loss of expression of PAB4 or any eIF4G isoform individually had little to no observable effect on plant growth or fertility [33, 36], repeated attempts to isolate a *pab4 eif4g* double mutant failed. As the *pab2 eif4g*, *pab8 eif4g*, and *pab2 pab8 eif4g* mutants were viable, this suggesting synthetic lethality between *pab4* and *eif4g*. The requirement for co-expression of PAB4 and eIF4G was sensitive to gene copy number in that neither *pab4/PAB4 eif4g/eif4g* nor *pab4/pab4 eif4g/eIF4G* could be isolated. Isolation of a *pab4/PAB4 eif4g/eIF4G* double heterozygous mutant was possible although the plants were characterized by reduced chlorophyll content and very poor fertility. Reciprocal crosses between *pab4/PAB4 eif4g/eIF4G* and WT plants revealed that most ovaries and pollen from the *pab4/PAB4 eif4g/eIF4G* parent involved in successful fertilization events were genotypically *PAB4 eif4g*. The presence of *pab4 eif4g* ovaries, although few in number, demonstrated that ovary development can occur in the absence of PAB4 and eIF4G co-expression. However, out of a combined total of 161 progeny examined, no successful fertilization events resulted from *pab4 eif4g* pollen, suggesting that their co-expression may be needed for male gametophyte development or function. The lack of *pab4 eif4g* pollen prevents the generation of *pab4 eif4g* double mutant embryos. Consequently, it is not possible to conclude that *PAB4* and *eIF4G* co-expression is essential for embryo development or for vegetative growth. However, the poor fertility and reduced chlorophyll content exhibited by *pab4/PAB4 eif4g/eIF4G* plants does suggest that co-expression of PAB4 and eIF4G may be necessary to support other aspects of reproduction and normal vegetative growth.

As observed for eIF4G, repeated attempts to isolate a *pab4 eifiso4g2* combinatorial mutant failed, suggesting that loss of PAB4 and eIFiso4G2 expression results in synthetic lethality. The observed synthetic lethality was specific for eIFiso4G2 as the *pab4 eifiso4g1* double mutant was viable and its growth was similar to WT plants. Isolation of a *pab4/PAB4 eifiso4g2/eIFiso4G2* double heterozygous mutant was possible and these plants appeared similar to WT plants in their growth. Crosses between *pab4/PAB4 eifiso4g2/eIFiso4G2* and WT plants revealed that most ovaries from the *pab4/PAB4 eifiso4g2/eIFiso4G2* parent involved in successful fertilization events were predominantly *PAB4 eifiso4g2* genotypically but that ovaries that were *pab4 eIFiso4G2* were well represented. Similar results were obtained when *pab4/PAB4 eifiso4g2/eIFiso4G2* plants were used as the pollen donor in a cross with WT plants although *PAB4 eifiso4g2* and *pab4 eIFiso4G2* progeny were more equally represented. The presence of *pab4 eifiso4g2* ovaries and pollen, although few in number, suggests that co-expression of PAB4 and eIFiso4G2 is not essential for male or female gametophyte development, at least in the presence of eIFiso4G1 expression. Therefore, the failure to isolate *pab4 eifiso4g2* plants suggests that co-expression of PAB4 and eIFiso4G2 is needed to support either embryo development and/or vegetative growth.

Although eIFiso4G1 and eIFiso4G2 might be expected to exhibit substantial functional overlap as they are isoforms, eIFiso4G2 differs from eIFiso4G1 in that it evolved recently within the Brassicaceae and the Cleomaceae [19] but exhibits substantial sequence divergence from eIFiso4G1, particularly within those regions that function as binding sites for partner proteins and RNA [19]. eIFiso4G1 and eIFiso4G2 also exhibit distinct differences in translational preferences in that eIFiso4G2 is more functionally similar to eIF4G during translation than it is to eIFiso4G1 [19], supporting the synthetic lethality exhibited between *pab4* and *eif4g* and between *pab4* and *eifiso4g2*.

The combinatorial loss of expression from *eIF4G* and either *PAB2* or *PAB8* resulted in little to no decrease in fertility in the homozygous mutant state and the modestly reduced fertility of *pab2 pab8 eif4g* triple mutant plants was not greater than that observed for *pab2 pab8* plants [33]. *pab2/PAB2 eif4g/eIF4G* and *pab8/PAB8 eif4g/eIF4G* double heterozygous plants exhibited only a small reduction in ovaries and seed per silique in relation to that observed for *eif4g/eIF4G* plants, suggesting that the genetic interaction between *eIF4G* and *PAB2* and/or *PAB8* contributes modestly at best to fertility. Interestingly, loss of expression from either *PAB2* or *PAB8* in *pab4/PAB4 eif4g/eIF4G* plants partially reversed the defects in fertility observed for the latter, i.e., few successful and shorter siliques, fewer ovaries per silique, and reduced seed set. Loss of expression from either *PAB2* or *PAB8* also reversed the growth defects of *pab4/PAB4 eif4g/eIF4G* plants. This suggests a genetic interaction in which expression from either *PAB2* or *PAB8* exacerbates the effects of *PAB4* mutations in *pab4/PAB4 eif4g/eIF4G* plants which is relieved in the absence of their expression.

*PAB8* is the most recently evolved member of class II *PAB* genes and is largely limited to species of the Brassicaceae. As PAB8 protein is most similar to PAB2, it likely arose from a gene duplication of *PAB2* [25]. The combinatorial loss of expression from *PAB2* and *PAB8* results in a modest reduction in growth, a reduction in inflorescence internode length, and modest reductions in ovary and seed per silique [33]. The inflorescence of *pab2 pab8 eif4g* triple mutant plants were similar to those of *pab2 pab8* double mutants and its vegetative growth was similar, data suggesting little genetic interaction between these class II proteins and eIF4G. In contrast, *pab2 pab8 eifiso4g1/2* quadruple mutant plants were smaller than *eifiso4g1/2* double mutant plants, the latter of which are characterized by a substantial reduction in stature, reduced chlorophyll content, delayed vegetative-to-floral transition, and poor fertility [33, 34, 36]. In addition to a further reduction in stature, the delay in the vegetative-to-floral transition in *pab2 pab8 eifiso4g1/2* mutants was more pronounced and fertility was extremely poor compared to *eifiso4g1/2* plants, resulting in only a few seed per plant. These results suggest that the effects of loss of expression from *PAB2* and *PAB8* and the loss of expression from *eIFiso4G1* and *eIFiso4G2* may be additive.

In addition to the synthetic lethality between *pab4* and *eif4g*, *pab4/PAB4 eif4g/eIF4G* plants exhibited non-Mendelian inheritance of the mutant loci. Progeny from *pab4/PAB4 eif4g/eIF4G* plants were largely *PAB4/PAB4 eif4g/eif4g* (67.5% versus 6.25% expected) or *PAB4/PAB4 eif4g/eIF4G* (23.8% versus 12.5% expected) along with *pab4/pab4 eIF4G/eIF4G* (5.0% versus 6.25% expected) and *pab4/PAB4 eif4g/eIF4G* (3.75% versus 25.0% expected). Thus, progeny containing the two *PAB4* alleles as wild-type in the presence of *eif4g* in either a homozygous or heterozygous state was preferred. No *pab4/pab4 eif4g/eif4g* plants were obtained consistent with the lack of *pab4 eif4g* pollen participating in successful fertilization events. Interestingly, no *PAB4/PAB4 eIF4G/eIF4G* plants were obtained. Reciprocal crosses with WT plants revealed that 93.0% of the eggs or 93.6% of the pollen from *pab4/PAB4 eif4g/eIF4G* plants involved in successful fertilization and seed development were genotypically *PAB4 eif4g*, consistent with the over-representation (67.5% versus 6.25% expected) of *PAB4/PAB4 eif4g/eif4g* progeny from selfed *pab4/PAB4 eif4g/eIF4G* plants. The low-frequency occurrence of *pab4 eif4g* eggs involved in successful fertilization and seed development demonstrated that co-expression of PAB4 and eIF4G was not required for female gametophyte development and/or function but that the absence of *pab4 eif4g* pollen involved in successful fertilization and seed development suggests co-expression of PAB4 and eIF4G may be necessary for male gametophyte development and/or function. Combining null mutations affecting eIF4E1 and eIFiso4E in *Arabidopsis* was lethal in that transmission through the male gametophyte failed although transmission through the female gametophyte was successful [37], supporting the conclusion that pollen development or function is affected to a greater extent by loss of critical components of the translational machinery than is egg development/function.

The predominance of *PAB4 eif4g* eggs and pollen and the lack of *PAB4 eIF4G* eggs or the very few *PAB4 eIF4G* pollen in a *pab4/PAB4 eif4g/eIF4G* parent participating in successful fertilization events suggests possible defects including alterations to selection of the megaspore (which gives rise to the female gametophyte), pollen tube guidance, or rate of pollen tube growth. The observation that the eggs and pollen from a *pab4/PAB4* or an *eif4g/eIF4G* parent that successfully participated in fertilization events were not biased to the same extent as that observed in a *pab4/PAB4 eif4g/eIF4G* parent suggests that it is the interaction of the mutant or wild-type alleles of *PAB4* and *eIF4G* that is responsible for altered genotypic distribution of eggs and pollen in a *pab4/PAB4 eif4g/eIF4G* parent.

The absence of *PAB2* or *PAB8* expression in *pab4/PAB4 eif4g/eIF4G* plants altered the genotype of the eggs and pollen participating in successful fertilization events from being predominantly *PAB4 eif4g* in a *pab4/PAB4 eif4g/eIF4G* parent to a mix of *PAB4 eif4g* and *pab4 eIF4G* eggs and pollen. These results suggest that *PAB2* or *PAB8* expression contributes to the predominance of *PAB4 eif4g* eggs and pollen in a *pab4/PAB4 eif4g/eIF4G* parent that are involved in successful fertilization events, indicating a degree of complexity in the genetic interaction between class II *PAB* genes and *eIF4G* during reproduction.

Synthetic lethality was also observed between *pab4* and *eifiso4g2*. *pab4/PAB4 eifiso4g2/eIFiso4G2* plants exhibited non-Mendelian inheritance of the mutant loci although to a lesser extent than that observed in *pab4/PAB4 eif4g/eIF4G* plants. Reciprocal crosses with WT plants revealed that the eggs and pollen participating in successful fertilization events were predominantly *PAB4 eif4iso4g2* or *pab4 eIFiso4G2* although *pab4 eifiso4g2* eggs and pollen also participated, indicating that expression from *PAB4* and *eIFiso4G2* is not essential for male or female gametophyte development and function. It is possible that expression from *eIFiso4G1* may account for this difference. As in *pab4/PAB4 eif4g/eIF4G* plants, eggs and pollen from *pab4/PAB4 eifiso4g2/eIFiso4G2* parents that were genotypically *PAB4 eIFiso4G2* were under represented. In contrast, however, loss of *PAB2* or *PAB8* expression in *pab4/PAB4 eifiso4g2/eifiso4g2* parents did not alter the genotypic distribution of eggs and pollen participating in successful fertilization events to the same extent as observed in *pab4/PAB4 eif4g/eIF4G* plants.

## Conclusion

The synthetic lethality between *pab4* and *eif4g* and between *pab4* and *eifiso4g2* suggests that the functional interaction between PAB4 and eIF4G may be similar to that between PAB4 and eIFiso4G2 and these interactions may target similar pathways. Whether these genetic interactions affect global protein synthesis or are of particular importance for expression from specific genes is unknown. The reduced chlorophyll content but relatively normal vegetative growth of *pab4/PAB4 eif4g/eIF4G* plants, however, suggests that expression from specific genes involved in chloroplast development or function may be dependent on a functional interaction between PAB4 and eIF4G. Similarly, the observed participation of *pab4 eif4g* ovaries but not *pab4 eif4g* pollen in crosses resulting in non-Mendelian inheritance suggests that the functional interaction between PAB4 and eIF4G may be important for the expression of genes involved specifically in aspects of pollen development or function. In contrast, the observed reduction in vegetative growth of *pab2/8 eifiso4g1/2* quadruple mutants may indicate effects on global protein synthesis. Future work investigating the basis for the synthetic lethality between *pab4* and *eif4g* or between *pab4* and *eifiso4g2* as well as how these mutant combinations affect male or female gametophyte development in double heterozygous parents will be needed to reveal those pathways that require co-expression of PAB4 and eIF4G (or eIFiso4G2).

## Supporting information

S1 Fig Silique, embryo, and flower development of *pab* and *eif4g* combinatorial mutants. Dissected siliques showing developing embryos (top panels) and flowers (bottom panels) are shown for wild-type and each *pab*/*eif4g* combinatorial mutant. Bars represent 5 mm.
(EPS)

S2 Fig Flower, silique, and embryo development of class II *pab* heterozygous mutants. Dissected siliques showing developing flowers (top panels) and embryos (bottom panels) are shown for wild-type and each *pab* heterozygous mutant. Bars represent 5 mm.
(EPS)

S3 Fig Silique, embryo, and flower development of *eif4g* and *eifiso4g* mutants. Dissected siliques showing developing embryos (top panels) and flowers (bottom panels) are shown for wild-type, *eif4g*, and *eifiso4g* mutants. Bars represent 5 mm.
(EPS)

S4 Fig Pollen viability staining of anthers just prior to dehiscence. Viability was examined in (A) WT; (B) *pab4*; (C) *eif4g*; (D) *PAB4*+/- *eIF4G*+/- flowers in which viable pollen stain magenta and inviable pollen stain blue. Viability staining was performed as described in S1 Text.
(EPS)

S5 Fig Silique, embryo, and flower development of *pab* and *eifiso4g* combinatorial mutants. Dissected siliques showing developing embryos (top panels) and flowers (bottom panels) are shown for wild-type and *pab*/*eifiso4g* combinatorial mutants. Bars represent 5 mm.
(EPS)

S6 Fig Failure of PAB4+/- eifiso4g1 eIFiso4G2+/- progeny to grow. Growth of WT seedlings (left side) and progeny from selfed *PAB4*+/- *eifiso4g1 eIFiso4G2*+/- plants (right side) at (A) 7 days; (B) 9 days; (C) 11 days post germination. The panels show growth seedlings of WT seedlings over time while some progeny of *PAB4*+/- *eifiso4g1 eIFiso4G2*+/- plants fail to grow past the cotyledon stage and eventually die.
(EPS)

S1 Table Tukey HSD results of *eif4g* mutants for ovaries/silique. (DOCX)

S2 Table Tukey HSD results of *eif4g* mutants for siliques/plant. (DOCX)

S3 Table Tukey HSD results of *eif4g* mutants for silique lengths. (DOCX)

S4 Table Tukey HSD results of *eifiso4g* mutants for ovaries/silique. (DOCX)

S5 Table Tukey HSD results of *eifiso4g* mutants for siliques/plant. (DOCX)

S6 Table Tukey HSD results of *eifiso4g* mutants for silique lengths. (DOCX)

S7 Table Tukey HSD results of *eIFiso4G* heterozygous mutants for ovaries/silique. (DOCX)

S8 Table Tukey HSD results of *eIFiso4G* heterozygous mutants for siliques/plant. (DOCX)

S9 Table Tukey HSD results of *eIFiso4G* heterozygous mutants for silique lengths. (DOCX)

S1 Text Method for pollen viability assay. (DOCX)
