# Supplementary material for: Plant growth and fertility requires functional interactions between specific PABP and eIF4G gene family members
Source: PLoS One. 2018 Jan 30;13(1):e0191474. doi: 10.1371/journal.pone.0191474 (PMC5790229; doi:10.1371/journal.pone.0191474)
Supplement: S1 Table — (DOCX) [file pone.0191474.s007.docx]

**S1 Table. Tukey HSD results of *eif4g* mutants for ovaries/silique.**

| treatments  pair | Tukey HSD  Q statistic | Tukey HSD  p-value | Tukey HSD  inferfence |
| --- | --- | --- | --- |
| A vs B | 11.2862 | 0.0010053 | ** p<0.01 |
| A vs C | 1.7722 | 0.8999947 | insignificant |
| A vs D | 5.2391 | 0.0109347 | * p<0.05 |
| A vs E | 11.4032 | 0.0010053 | ** p<0.01 |
| A vs F | 17.1875 | 0.0010053 | ** p<0.01 |
| A vs G | 13.0723 | 0.0010053 | ** p<0.01 |
| A vs H | 18.5827 | 0.0010053 | ** p<0.01 |
| A vs I | 10.0953 | 0.0010053 | ** p<0.01 |
| A vs J | 7.6860 | 0.0010053 | ** p<0.01 |
| A vs K | 11.5585 | 0.0010053 | ** p<0.01 |
| B vs C | 9.7568 | 0.0010053 | ** p<0.01 |
| B vs D | 6.5079 | 0.0010053 | ** p<0.01 |
| B vs E | 0.1170 | 0.8999947 | insignificant |
| B vs F | 5.4404 | 0.0067249 | ** p<0.01 |
| B vs G | 1.2356 | 0.8999947 | insignificant |
| B vs H | 7.0538 | 0.0010053 | ** p<0.01 |
| B vs I | 1.4337 | 0.8999947 | insignificant |
| B vs J | 3.8430 | 0.1766355 | insignificant |
| B vs K | 0.6060 | 0.8999947 | insignificant |
| C vs D | 3.5137 | 0.2861299 | insignificant |
| C vs E | 9.8763 | 0.0010053 | ** p<0.01 |
| C vs F | 15.7412 | 0.0010053 | ** p<0.01 |
| C vs G | 11.2564 | 0.0010053 | ** p<0.01 |
| C vs H | 17.1884 | 0.0010053 | ** p<0.01 |
| C vs I | 8.5102 | 0.0010053 | ** p<0.01 |
| C vs J | 6.0467 | 0.0013990 | ** p<0.01 |
| C vs K | 9.9150 | 0.0010053 | ** p<0.01 |
| D vs E | 6.6297 | 0.0010053 | ** p<0.01 |
| D vs F | 12.4797 | 0.0010053 | ** p<0.01 |
| D vs G | 7.1475 | 0.0010053 | ** p<0.01 |
| D vs H | 14.0151 | 0.0010053 | ** p<0.01 |
| D vs I | 5.1651 | 0.0130143 | * p<0.05 |
| D vs J | 2.6528 | 0.6614561 | insignificant |
| D vs K | 6.4059 | 0.0010053 | ** p<0.01 |
| E vs F | 5.3187 | 0.0090421 | ** p<0.01 |
| E vs G | 1.3838 | 0.8999947 | insignificant |
| E vs H | 6.9343 | 0.0010053 | ** p<0.01 |
| E vs I | 1.5531 | 0.8999947 | insignificant |
| E vs J | 3.9625 | 0.1457322 | insignificant |
| E vs K | 0.7321 | 0.8999947 | insignificant |
| F vs G | 8.3983 | 0.0010053 | ** p<0.01 |
| F vs H | 1.7876 | 0.8999947 | insignificant |
| F vs I | 7.0625 | 0.0010053 | ** p<0.01 |
| F vs J | 9.5747 | 0.0010053 | ** p<0.01 |
| F vs K | 6.5173 | 0.0010053 | ** p<0.01 |
| G vs H | 10.3418 | 0.0010053 | ** p<0.01 |
| G vs I | 0.5628 | 0.8999947 | insignificant |
| G vs J | 3.6583 | 0.2338184 | insignificant |
| G vs K | 0.5782 | 0.8999947 | insignificant |
| H vs I | 8.6782 | 0.0010053 | ** p<0.01 |
| H vs J | 11.1416 | 0.0010053 | ** p<0.01 |
| H vs K | 8.2368 | 0.0010053 | ** p<0.01 |
| I vs J | 2.4635 | 0.7421460 | insignificant |
| I vs K | 0.9278 | 0.8999947 | insignificant |

**A = WT**

**B = *pab2*+/-**

**C = *pab4*+/-**

**D = *pab8*+/-**

**E = *eif4g*+/-**

**F = *pab2*+/- *eif4g*+/-**

**G = *pab4*+/- *eif4g*+/-**

**H = *pab8*+/- *eif4g*+/-**

**I = *pab2*+/- *pab8*+/- *eif4g*+/-**

**J = *pab2* *pab4*+/- *eif4g*+/-**

**K = *pab8* *pab4*+/- *eif4g*+/-**
