# Supplementary material for: Plant growth and fertility requires functional interactions between specific PABP and eIF4G gene family members
Source: PLoS One. 2018 Jan 30;13(1):e0191474. doi: 10.1371/journal.pone.0191474 (PMC5790229; doi:10.1371/journal.pone.0191474)
Supplement: S2 Table — (DOCX) [file pone.0191474.s008.docx]

**S2 Table. Tukey HSD results of *eif4g* mutants for siliques/plant.**

| treatments  pair | Tukey HSD  Q statistic | Tukey HSD  p-value | Tukey HSD  inferfence |
| --- | --- | --- | --- |
| A vs B | 0.5448 | 0.8999947 | insignificant |
| A vs C | 0.1702 | 0.8999947 | insignificant |
| A vs D | 0.1702 | 0.8999947 | insignificant |
| A vs E | 0.2383 | 0.8999947 | insignificant |
| A vs F | 0.9534 | 0.8999947 | insignificant |
| A vs G | 21.3485 | 0.0010053 | ** p<0.01 |
| A vs H | 0.8853 | 0.8999947 | insignificant |
| A vs I | 0.2383 | 0.8999947 | insignificant |
| A vs J | 4.9543 | 0.0485937 | * p<0.05 |
| A vs K | 4.1872 | 0.1446613 | insignificant |
| B vs C | 0.3503 | 0.8999947 | insignificant |
| B vs D | 0.3503 | 0.8999947 | insignificant |
| B vs E | 0.7325 | 0.8999947 | insignificant |
| B vs F | 0.3822 | 0.8999947 | insignificant |
| B vs G | 19.4601 | 0.0010053 | ** p<0.01 |
| B vs H | 0.3185 | 0.8999947 | insignificant |
| B vs I | 0.2866 | 0.8999947 | insignificant |
| B vs J | 3.9811 | 0.1887897 | insignificant |
| B vs K | 3.2533 | 0.4275903 | insignificant |
| C vs D | 0.0000 | 0.8999947 | insignificant |
| C vs E | 0.3822 | 0.8999947 | insignificant |
| C vs F | 0.7325 | 0.8999947 | insignificant |
| C vs G | 19.8104 | 0.0010053 | ** p<0.01 |
| C vs H | 0.6688 | 0.8999947 | insignificant |
| C vs I | 0.0637 | 0.8999947 | insignificant |
| C vs J | 4.3728 | 0.1128858 | insignificant |
| C vs K | 3.6609 | 0.2773679 | insignificant |
| D vs E | 0.3822 | 0.8999947 | insignificant |
| D vs F | 0.7325 | 0.8999947 | insignificant |
| D vs G | 19.8104 | 0.0010053 | ** p<0.01 |
| D vs H | 0.6688 | 0.8999947 | insignificant |
| D vs I | 0.0637 | 0.8999947 | insignificant |
| D vs J | 4.3728 | 0.1128858 | insignificant |
| D vs K | 3.6609 | 0.2773679 | insignificant |
| E vs F | 1.1147 | 0.8999947 | insignificant |
| E vs G | 20.1926 | 0.0010053 | ** p<0.01 |
| E vs H | 1.0510 | 0.8999947 | insignificant |
| E vs I | 0.4459 | 0.8999947 | insignificant |
| E vs J | 4.8001 | 0.0612669 | insignificant |
| E vs K | 4.1056 | 0.1612541 | insignificant |
| F vs G | 19.0779 | 0.0010053 | ** p<0.01 |
| F vs H | 0.0637 | 0.8999947 | insignificant |
| F vs I | 0.6688 | 0.8999947 | insignificant |
| F vs J | 3.5538 | 0.3124605 | insignificant |
| F vs K | 4.1056 | 0.1612541 | insignificant |
| G vs H | 19.1416 | 0.0010053 | ** p<0.01 |
| G vs I | 19.7467 | 0.0010053 | ** p<0.01 |
| G vs J | 17.7760 | 0.0010053 | ** p<0.01 |
| G vs K | 19.3866 | 0.0010053 | ** p<0.01 |
| H vs I | 0.6051 | 0.8999947 | insignificant |
| H vs J | 3.6250 | 0.2888972 | insignificant |
| H vs K | 2.8828 | 0.5737285 | insignificant |
| I vs J | 4.3016 | 0.1245516 | insignificant |
| I vs K | 3.5868 | 0.3014148 | insignificant |

**A = WT**

**B = *pab2*+/-**

**C = *pab4*+/-**

**D = *pab8*+/-**

**E = *eif4g*+/-**

**F = *pab2*+/- *eif4g*+/-**

**G = *pab4*+/- *eif4g*+/-**

**H = *pab8*+/- *eif4g*+/-**

**I = *pab2*+/- *pab8*+/- *eif4g*+/-**

**J = *pab2* *pab4*+/- *eif4g*+/-**

**K = *pab8* *pab4*+/- *eif4g*+/-**
