# Supplementary material for: Plant growth and fertility requires functional interactions between specific PABP and eIF4G gene family members
Source: PLoS One. 2018 Jan 30;13(1):e0191474. doi: 10.1371/journal.pone.0191474 (PMC5790229; doi:10.1371/journal.pone.0191474)
Supplement: S3 Table — (DOCX) [file pone.0191474.s009.docx]

**S3 Table. Tukey HSD results of *eif4g* mutants for silique lengths.**

| treatments  pair | Tukey HSD  Q statistic | Tukey HSD  p-value | Tukey HSD  inferfence |
| --- | --- | --- | --- |
| A vs B | 11.7873 | 0.0010053 | ** p<0.01 |
| A vs C | 2.6991 | 0.6417667 | insignificant |
| A vs D | 1.6741 | 0.8999947 | insignificant |
| A vs E | 5.6798 | 0.0037713 | ** p<0.01 |
| A vs F | 9.2441 | 0.0010053 | ** p<0.01 |
| A vs G | 35.4747 | 0.0010053 | ** p<0.01 |
| A vs H | 8.5647 | 0.0010053 | ** p<0.01 |
| A vs I | 7.4196 | 0.0010053 | ** p<0.01 |
| A vs J | 1.6741 | 0.8999947 | insignificant |
| A vs K | 2.0734 | 0.8999947 | insignificant |
| B vs C | 14.0264 | 0.0010053 | ** p<0.01 |
| B vs D | 9.7920 | 0.0010053 | ** p<0.01 |
| B vs E | 5.6935 | 0.0036436 | ** p<0.01 |
| B vs F | 1.9918 | 0.8999947 | insignificant |
| B vs G | 21.9272 | 0.0010053 | ** p<0.01 |
| B vs H | 2.8967 | 0.5576668 | insignificant |
| B vs I | 4.4324 | 0.0632779 | insignificant |
| B vs J | 9.7920 | 0.0010053 | ** p<0.01 |
| B vs K | 10.2465 | 0.0010053 | ** p<0.01 |
| C vs D | 4.2344 | 0.0912365 | insignificant |
| C vs E | 8.0708 | 0.0010053 | ** p<0.01 |
| C vs F | 11.4844 | 0.0010053 | ** p<0.01 |
| C vs G | 36.9787 | 0.0010053 | ** p<0.01 |
| C vs H | 10.8675 | 0.0010053 | ** p<0.01 |
| C vs I | 9.8338 | 0.0010053 | ** p<0.01 |
| C vs J | 4.2344 | 0.0912365 | insignificant |
| C vs K | 4.8108 | 0.0291262 | * p<0.05 |
| D vs E | 3.9155 | 0.1582470 | insignificant |
| D vs F | 7.4161 | 0.0010053 | ** p<0.01 |
| D vs G | 32.4349 | 0.0010053 | ** p<0.01 |
| D vs H | 6.7123 | 0.0010053 | ** p<0.01 |
| D vs I | 5.5270 | 0.0055413 | ** p<0.01 |
| D vs J | 0.0000 | 0.8999947 | insignificant |
| D vs K | 0.2652 | 0.8999947 | insignificant |
| E vs F | 3.5206 | 0.2840025 | insignificant |
| E vs G | 27.5497 | 0.0010053 | ** p<0.01 |
| E vs H | 2.7464 | 0.6216406 | insignificant |
| E vs I | 1.4403 | 0.8999947 | insignificant |
| E vs J | 3.9155 | 0.1582470 | insignificant |
| E vs K | 3.9406 | 0.1517556 | insignificant |
| F vs G | 23.0702 | 0.0010053 | ** p<0.01 |
| F vs H | 0.8297 | 0.8999947 | insignificant |
| F vs I | 2.2297 | 0.8415767 | insignificant |
| F vs J | 7.4161 | 0.0010053 | ** p<0.01 |
| F vs K | 7.6962 | 0.0010053 | ** p<0.01 |
| G vs H | 24.5569 | 0.0010053 | ** p<0.01 |
| G vs I | 27.1293 | 0.0010053 | ** p<0.01 |
| G vs J | 32.4349 | 0.0010053 | ** p<0.01 |
| G vs K | 34.6247 | 0.0010053 | ** p<0.01 |
| H vs I | 1.4024 | 0.8999947 | insignificant |
| H vs J | 6.7123 | 0.0010053 | ** p<0.01 |
| H vs K | 6.9410 | 0.0010053 | ** p<0.01 |
| I vs J | 5.5270 | 0.0055413 | ** p<0.01 |
| I vs K | 5.6669 | 0.0038852 | ** p<0.01 |

**A = WT**

**B = *pab2*+/-**

**C = *pab4*+/-**

**D = *pab8*+/-**

**E = *eif4g*+/-**

**F = *pab2*+/- *eif4g*+/-**

**G = *pab4*+/- *eif4g*+/-**

**H = *pab8*+/- *eif4g*+/-**

**I = *pab2*+/- *pab8*+/- *eif4g*+/-**

**J = *pab2* *pab4*+/- *eif4g*+/-**

**K = *pab8* *pab4*+/- *eif4g*+/-**
