# Supplementary material for: Plant growth and fertility requires functional interactions between specific PABP and eIF4G gene family members
Source: PLoS One. 2018 Jan 30;13(1):e0191474. doi: 10.1371/journal.pone.0191474 (PMC5790229; doi:10.1371/journal.pone.0191474)
Supplement: S4 Table — (DOCX) [file pone.0191474.s010.docx]

**S4 Table. Tukey HSD results of *eifiso4g* mutants for ovaries/silique.**

| treatments  pair | Tukey HSD  Q statistic | Tukey HSD  p-value | Tukey HSD  inferfence |
| --- | --- | --- | --- |
| A vs B | 2.7762 | 0.4479486 | insignificant |
| A vs C | 4.7012 | 0.0217401 | * p<0.05 |
| A vs D | 3.1267 | 0.3014260 | insignificant |
| A vs E | 3.8247 | 0.1104715 | insignificant |
| A vs F | 0.7228 | 0.8999947 | insignificant |
| A vs G | 22.7593 | 0.0010053 | ** p<0.01 |
| B vs C | 7.7058 | 0.0010053 | ** p<0.01 |
| B vs D | 0.3049 | 0.8999947 | insignificant |
| B vs E | 1.0721 | 0.8999947 | insignificant |
| B vs F | 2.1551 | 0.7039180 | insignificant |
| B vs G | 20.3639 | 0.0010053 | ** p<0.01 |
| C vs D | 8.1759 | 0.0010053 | ** p<0.01 |
| C vs E | 8.7992 | 0.0010053 | ** p<0.01 |
| C vs F | 5.6652 | 0.0025199 | ** p<0.01 |
| C vs G | 28.8083 | 0.0010053 | ** p<0.01 |
| D vs E | 0.7885 | 0.8999947 | insignificant |
| D vs F | 2.5107 | 0.5591264 | insignificant |
| D vs G | 20.4824 | 0.0010053 | ** p<0.01 |
| E vs F | 3.2484 | 0.2581309 | insignificant |
| E vs G | 19.2514 | 0.0010053 | ** p<0.01 |
| F vs G | 23.0391 | 0.0010053 | ** p<0.01 |

**A = WT**

**B = *eifiso4g1***

**C = *pab4* *eifiso4g1***

**D = *eifiso4g2***

**E = *pab2 eifiso4g2***

**F = *pab8 eifiso4g2***

**G = *eifiso4g1/2***
