# Supplementary material for: Plant growth and fertility requires functional interactions between specific PABP and eIF4G gene family members
Source: PLoS One. 2018 Jan 30;13(1):e0191474. doi: 10.1371/journal.pone.0191474 (PMC5790229; doi:10.1371/journal.pone.0191474)
Supplement: S5 Table — (DOCX) [file pone.0191474.s011.docx]

**S5 Table. Tukey HSD results of *eifiso4g* mutants for siliques/plant.**

| treatments  pair | Tukey HSD  Q statistic | Tukey HSD  p-value | Tukey HSD  inferfence |
| --- | --- | --- | --- |
| A vs B | 1.7442 | 0.8605302 | insignificant |
| A vs C | 2.2372 | 0.6735808 | insignificant |
| A vs D | 0.8721 | 0.8999947 | insignificant |
| A vs E | 0.4360 | 0.8999947 | insignificant |
| A vs F | 1.8895 | 0.8054119 | insignificant |
| A vs G | 17.2322 | 0.0010053 | ** p<0.01 |
| B vs C | 0.3270 | 0.8999947 | insignificant |
| B vs D | 0.8158 | 0.8999947 | insignificant |
| B vs E | 2.0394 | 0.7485705 | insignificant |
| B vs F | 0.1360 | 0.8999947 | insignificant |
| B vs G | 13.8468 | 0.0010053 | ** p<0.01 |
| C vs D | 1.1991 | 0.8999947 | insignificant |
| C vs E | 2.5072 | 0.5711599 | insignificant |
| C vs F | 0.1817 | 0.8999947 | insignificant |
| C vs G | 14.7815 | 0.0010053 | ** p<0.01 |
| D vs E | 1.2236 | 0.8999947 | insignificant |
| D vs F | 0.9517 | 0.8999947 | insignificant |
| D vs G | 14.7888 | 0.0010053 | ** p<0.01 |
| E vs F | 2.1754 | 0.6970157 | insignificant |
| E vs G | 16.2017 | 0.0010053 | ** p<0.01 |
| F vs G | 13.6898 | 0.0010053 | ** p<0.01 |

**A = WT**

**B = *eifiso4g1***

**C = *pab4* *eifiso4g1***

**D = *eifiso4g2***

**E = *pab2 eifiso4g2***

**F = *pab8 eifiso4g2***

**G = *eifiso4g1/2***
