# Supplementary material for: Plant growth and fertility requires functional interactions between specific PABP and eIF4G gene family members
Source: PLoS One. 2018 Jan 30;13(1):e0191474. doi: 10.1371/journal.pone.0191474 (PMC5790229; doi:10.1371/journal.pone.0191474)
Supplement: S6 Table — (DOCX) [file pone.0191474.s012.docx]

**S6 Table. Tukey HSD results of *eifiso4g* mutants for silique lengths.**

| treatments  pair | Tukey HSD  Q statistic | Tukey HSD  p-value | Tukey HSD  inferfence |
| --- | --- | --- | --- |
| A vs B | 3.8242 | 0.1077025 | insignificant |
| A vs C | 7.1839 | 0.0010053 | ** p<0.01 |
| A vs D | 1.2788 | 0.8999947 | insignificant |
| A vs E | 0.9198 | 0.8999947 | insignificant |
| A vs F | 2.7367 | 0.4639092 | insignificant |
| A vs G | 42.2016 | 0.0010053 | ** p<0.01 |
| B vs C | 10.7645 | 0.0010053 | ** p<0.01 |
| B vs D | 2.5293 | 0.5507176 | insignificant |
| B vs E | 2.8765 | 0.4019432 | insignificant |
| B vs F | 1.1803 | 0.8999947 | insignificant |
| B vs G | 36.7635 | 0.0010053 | ** p<0.01 |
| C vs D | 8.3458 | 0.0010053 | ** p<0.01 |
| C vs E | 7.9869 | 0.0010053 | ** p<0.01 |
| C vs F | 9.9206 | 0.0010053 | ** p<0.01 |
| C vs G | 49.5937 | 0.0010053 | ** p<0.01 |
| D vs E | 0.3533 | 0.8999947 | insignificant |
| D vs F | 1.4134 | 0.8999947 | insignificant |
| D vs G | 40.1614 | 0.0010053 | ** p<0.01 |
| E vs F | 1.7724 | 0.8600851 | insignificant |
| E vs G | 40.5304 | 0.0010053 | ** p<0.01 |
| F vs G | 39.3855 | 0.0010053 | ** p<0.01 |

**A = WT**

**B = *eifiso4g1***

**C = *pab4* *eifiso4g1***

**D = *eifiso4g2***

**E = *pab2 eifiso4g2***

**F = *pab8 eifiso4g2***

**G = *eifiso4g1/2***
