# Supplementary material for: Plant growth and fertility requires functional interactions between specific PABP and eIF4G gene family members
Source: PLoS One. 2018 Jan 30;13(1):e0191474. doi: 10.1371/journal.pone.0191474 (PMC5790229; doi:10.1371/journal.pone.0191474)
Supplement: S7 Table — (DOCX) [file pone.0191474.s013.docx]

**S7 Table. Tukey HSD results of *eIFiso4G* heterozygous mutants for ovaries/silique.**

| treatments  pair | Tukey HSD  Q statistic | Tukey HSD  p-value | Tukey HSD  inferfence |
| --- | --- | --- | --- |
| A vs B | 4.3981 | 0.0483327 | * p<0.05 |
| A vs C | 2.3380 | 0.6912746 | insignificant |
| A vs D | 5.1792 | 0.0093015 | ** p<0.01 |
| A vs E | 14.9433 | 0.0010053 | ** p<0.01 |
| A vs F | 4.6894 | 0.0270139 | * p<0.05 |
| A vs G | 0.6980 | 0.8999947 | insignificant |
| A vs H | 13.6483 | 0.0010053 | ** p<0.01 |
| B vs C | 2.1139 | 0.7840205 | insignificant |
| B vs D | 0.9081 | 0.8999947 | insignificant |
| B vs E | 11.2413 | 0.0010053 | ** p<0.01 |
| B vs F | 0.3871 | 0.8999947 | insignificant |
| B vs G | 3.9443 | 0.1098916 | insignificant |
| B vs H | 9.8608 | 0.0010053 | ** p<0.01 |
| C vs D | 2.9676 | 0.4258620 | insignificant |
| C vs E | 13.1451 | 0.0010053 | ** p<0.01 |
| C vs F | 2.4559 | 0.6424712 | insignificant |
| C vs G | 1.7566 | 0.8999947 | insignificant |
| C vs H | 11.7904 | 0.0010053 | ** p<0.01 |
| D vs E | 10.1231 | 0.0010053 | ** p<0.01 |
| D vs F | 0.5117 | 0.8999947 | insignificant |
| D vs G | 4.7787 | 0.0224189 | * p<0.05 |
| D vs H | 8.7684 | 0.0010053 | ** p<0.01 |
| E vs F | 10.6441 | 0.0010053 | ** p<0.01 |
| E vs G | 15.1856 | 0.0010053 | ** p<0.01 |
| E vs H | 1.3805 | 0.8999947 | insignificant |
| F vs G | 4.2576 | 0.0631587 | insignificant |
| F vs H | 9.2894 | 0.0010053 | ** p<0.01 |
| G vs H | 13.8051 | 0.0010053 | ** p<0.01 |

**A = WT**

**B = *pab2*+/- *eifiso4g2*+/-**

**C = *pab4*+/- *eifiso4g2*+/-**

**D = *pab8*+/- *eifiso4g2*+/-**

**E = *eifiso4g1*+/-**

**F = *eifiso4g2*+/-**

**G = *pab4*+/- *eifiso4g1*+/-**

**H = *eifiso4g1/2*+/-**
