# Supplementary material for: Plant growth and fertility requires functional interactions between specific PABP and eIF4G gene family members
Source: PLoS One. 2018 Jan 30;13(1):e0191474. doi: 10.1371/journal.pone.0191474 (PMC5790229; doi:10.1371/journal.pone.0191474)
Supplement: S8 Table — (DOCX) [file pone.0191474.s014.docx]

**S8 Table. Tukey HSD results of *eIFiso4G* heterozygous mutants for siliques/plant.**

| treatments  pair | Tukey HSD  Q statistic | Tukey HSD  p-value | Tukey HSD  inferfence |
| --- | --- | --- | --- |
| A vs B | 0.8000 | 0.8999947 | insignificant |
| A vs C | 2.7001 | 0.5554509 | insignificant |
| A vs D | 0.7000 | 0.8999947 | insignificant |
| A vs E | 0.3000 | 0.8999947 | insignificant |
| A vs F | 0.5000 | 0.8999947 | insignificant |
| A vs G | 2.4304 | 0.6573938 | insignificant |
| A vs H | 1.9001 | 0.8578155 | insignificant |
| B vs C | 1.7773 | 0.8999947 | insignificant |
| B vs D | 0.0935 | 0.8999947 | insignificant |
| B vs E | 0.4677 | 0.8999947 | insignificant |
| B vs F | 0.2806 | 0.8999947 | insignificant |
| B vs G | 1.4501 | 0.8999947 | insignificant |
| B vs H | 1.0290 | 0.8999947 | insignificant |
| C vs D | 1.8709 | 0.8688371 | insignificant |
| C vs E | 2.2451 | 0.7274209 | insignificant |
| C vs F | 2.0580 | 0.7981286 | insignificant |
| C vs G | 0.4500 | 0.8999947 | insignificant |
| C vs H | 0.7484 | 0.8999947 | insignificant |
| D vs E | 0.3742 | 0.8999947 | insignificant |
| D vs F | 0.1871 | 0.8999947 | insignificant |
| D vs G | 1.5501 | 0.8999947 | insignificant |
| D vs H | 1.1225 | 0.8999947 | insignificant |
| E vs F | 0.1871 | 0.8999947 | insignificant |
| E vs G | 1.9501 | 0.8389156 | insignificant |
| E vs H | 1.4967 | 0.8999947 | insignificant |
| F vs G | 1.7501 | 0.8999947 | insignificant |
| F vs H | 1.3096 | 0.8999947 | insignificant |
| G vs H | 0.3500 | 0.8999947 | insignificant |

**A = WT**

**B = *pab2*+/- *eifiso4g2*+/-**

**C = *pab4*+/- *eifiso4g2*+/-**

**D = *pab8*+/- *eifiso4g2*+/-**

**E = *eifiso4g1*+/-**

**F = *eifiso4g2*+/-**

**G = *pab4*+/- *eifiso4g1*+/-**

**H = *eifiso4g1/2*+/-**
