# Supplementary material for: Plant growth and fertility requires functional interactions between specific PABP and eIF4G gene family members
Source: PLoS One. 2018 Jan 30;13(1):e0191474. doi: 10.1371/journal.pone.0191474 (PMC5790229; doi:10.1371/journal.pone.0191474)
Supplement: S9 Table — (DOCX) [file pone.0191474.s015.docx]

**S9 Table. Tukey HSD results of *eIFiso4G* heterozygous mutants for silique lengths.**

| treatments  pair | Tukey HSD  Q statistic | Tukey HSD  p-value | Tukey HSD  inferfence |
| --- | --- | --- | --- |
| A vs B | 1.9341 | 0.8586772 | insignificant |
| A vs C | 2.2095 | 0.7443944 | insignificant |
| A vs D | 4.6904 | 0.0258677 | * p<0.05 |
| A vs E | 13.6779 | 0.0010053 | ** p<0.01 |
| A vs F | 6.0602 | 0.0010053 | ** p<0.01 |
| A vs G | 1.3447 | 0.8999947 | insignificant |
| A vs H | 18.8181 | 0.0010053 | ** p<0.01 |
| B vs C | 0.3054 | 0.8999947 | insignificant |
| B vs D | 2.7485 | 0.5207317 | insignificant |
| B vs E | 11.3964 | 0.0010053 | ** p<0.01 |
| B vs F | 4.0611 | 0.0877024 | insignificant |
| B vs G | 0.5802 | 0.8999947 | insignificant |
| B vs H | 16.5780 | 0.0010053 | ** p<0.01 |
| C vs D | 2.4021 | 0.6644941 | insignificant |
| C vs E | 10.8450 | 0.0010053 | ** p<0.01 |
| C vs F | 3.6851 | 0.1645549 | insignificant |
| C vs G | 0.8755 | 0.8999947 | insignificant |
| C vs H | 15.9705 | 0.0010053 | ** p<0.01 |
| D vs E | 8.2396 | 0.0010053 | ** p<0.01 |
| D vs F | 1.2419 | 0.8999947 | insignificant |
| D vs G | 3.3186 | 0.2780006 | insignificant |
| D vs H | 13.4896 | 0.0010053 | ** p<0.01 |
| E vs F | 7.0549 | 0.0010053 | ** p<0.01 |
| E vs G | 12.0166 | 0.0010053 | ** p<0.01 |
| E vs H | 6.1580 | 0.0010053 | ** p<0.01 |
| F vs G | 4.6412 | 0.0286824 | * p<0.05 |
| F vs H | 12.4519 | 0.0010053 | ** p<0.01 |
| G vs H | 17.1674 | 0.0010053 | ** p<0.01 |

**A = WT**

**B = *pab2*+/- *eifiso4g2*+/-**

**C = *pab4*+/- *eifiso4g2*+/-**

**D = *pab8*+/- *eifiso4g2*+/-**

**E = *eifiso4g1*+/-**

**F = *eifiso4g2*+/-**

**G = *pab4*+/- *eifiso4g1*+/-**

**H = *eifiso4g1/2*+/-**
