# Supplementary material for: Plant growth and fertility requires functional interactions between specific PABP and eIF4G gene family members
Source: PLoS One. 2018 Jan 30;13(1):e0191474. doi: 10.1371/journal.pone.0191474 (PMC5790229; doi:10.1371/journal.pone.0191474)
Supplement: S1 Text — (DOCX) [file pone.0191474.s016.docx]

**Supplemental Methods**

*Pollen viability assay*

The viability of pollen was examined essentially as described (Peterson et al., 2010). Briefly, stage 12 flower buds, in which the non-dehiscent anthers contained mature pollen, were fixed in Carnoy’s fixative (6 alcohol:3 chloroform:1 acetic acid) for 10 min and placed in stain solution [0.1 ml 95% alcohol, 0.01 ml Malachite green (1% solution in 95% alcohol), 0.55 ml water, 0.25 ml glycerol, 0.05 ml Acid fuchsin (1% solution in water), 0.005 ml Orange G (1% solution in water), 0.04 ml glacial acetic acid] for 2 hr. Stained buds were placed on a microscope slide and excess fixative was removed prior to placing a cover-slip over the sample. Pressure was applied to flatten the specimen and the specimens were examined under a Leitz microscope using a digital camera to record the images. Viable pollen stain magenta while non-viable pollen stain blue.
